# L Antigen Family Member 3 stabilizes stalled replication forks by reducing MRE11 resection to maintain genome stability

**DOI:** 10.1093/nar/gkag842

**Published:** 2026-08-24

**Authors:** Meng Lyu, Guihua Yang, Shaopeng Jia, Xiangze Zhang, Yingzheng Li, Yujing Feng, Xinxing Lyu

**Affiliations:** Dermatology Hospital of Shandong First Medical University, Jinan, Shandong 250022, P.R. China; Shandong Provincial Institute of Dermatology and Venereology, Shandong Academy of Medical Sciences, Jinan, Shandong 250022, P.R. China; School of Clinical and Basic Medical Sciences, Shandong First Medical University and Shandong Academy of Medical Sciences, Jinan, Shandong 250117, P.R. China; School of Clinical and Basic Medical Sciences, Shandong First Medical University and Shandong Academy of Medical Sciences, Jinan, Shandong 250117, P.R. China; School of Clinical and Basic Medical Sciences, Shandong First Medical University and Shandong Academy of Medical Sciences, Jinan, Shandong 250117, P.R. China; School of Clinical and Basic Medical Sciences, Shandong First Medical University and Shandong Academy of Medical Sciences, Jinan, Shandong 250117, P.R. China; School of Clinical and Basic Medical Sciences, Shandong First Medical University and Shandong Academy of Medical Sciences, Jinan, Shandong 250117, P.R. China; School of Laboratory Animal and Shandong Laboratory Animal Center, Shandong First Medical University and Shandong Academy of Medical Sciences, Jinan, Shandong 250117, P.R. China; Dermatology Hospital of Shandong First Medical University, Jinan, Shandong 250022, P.R. China; Shandong Provincial Institute of Dermatology and Venereology, Shandong Academy of Medical Sciences, Jinan, Shandong 250022, P.R. China; Science and Technology Innovation Center, Shandong First Medical University, Jinan, Shandong 250117, P.R. China

## Abstract

L Antigen Family Member 3 (LAGE3) is a core component of the KEOPS complex, mutations of which are implicated in Galloway–Mowat syndrome (GAMOS). The KEOPS complex is known to function in telomere maintenance and tRNA N6-threonylcarbamoyladenosine (t6A) modification. However, whether the KEOPS complex also contributes to chromosomal DNA regulation through additional mechanisms remains elusive. Here, we find that LAGE3 and the other four components of KEOPS localize to the stalled forks and protect the nascent DNA strand from degradation under DNA replication stress. TurboID-mediated proximity labeling identifies the MRE11 nuclease as a LAGE3-interacting partner, and LAGE3 deficiency leads to unscheduled stalled forks degradation in an MRE11 nuclease-dependent manner. Similarly, TP53RK, another KEOPS subunit, also exhibits a protective role at stalled forks. *In vitro* experiments demonstrate that the KEOPS complex prefers to bind to a ds-ssDNA junction, then directly limits MRE11 to degrade nascent DNA. In addition, GAMOS-associated LAGE3 mutations disrupt KEOPS complex assembly, preventing its recruitment to stalled replication forks and consequently leading to unscheduled fork resection and genome instability under DNA replication stress. Collectively, our findings reveal that LAGE3 acts as a stalled fork protector to prevent genome instability during DNA replication stress, providing new insights into GAMOS pathogenesis.

## Graphical Abstract

**Graphical Abstract:**
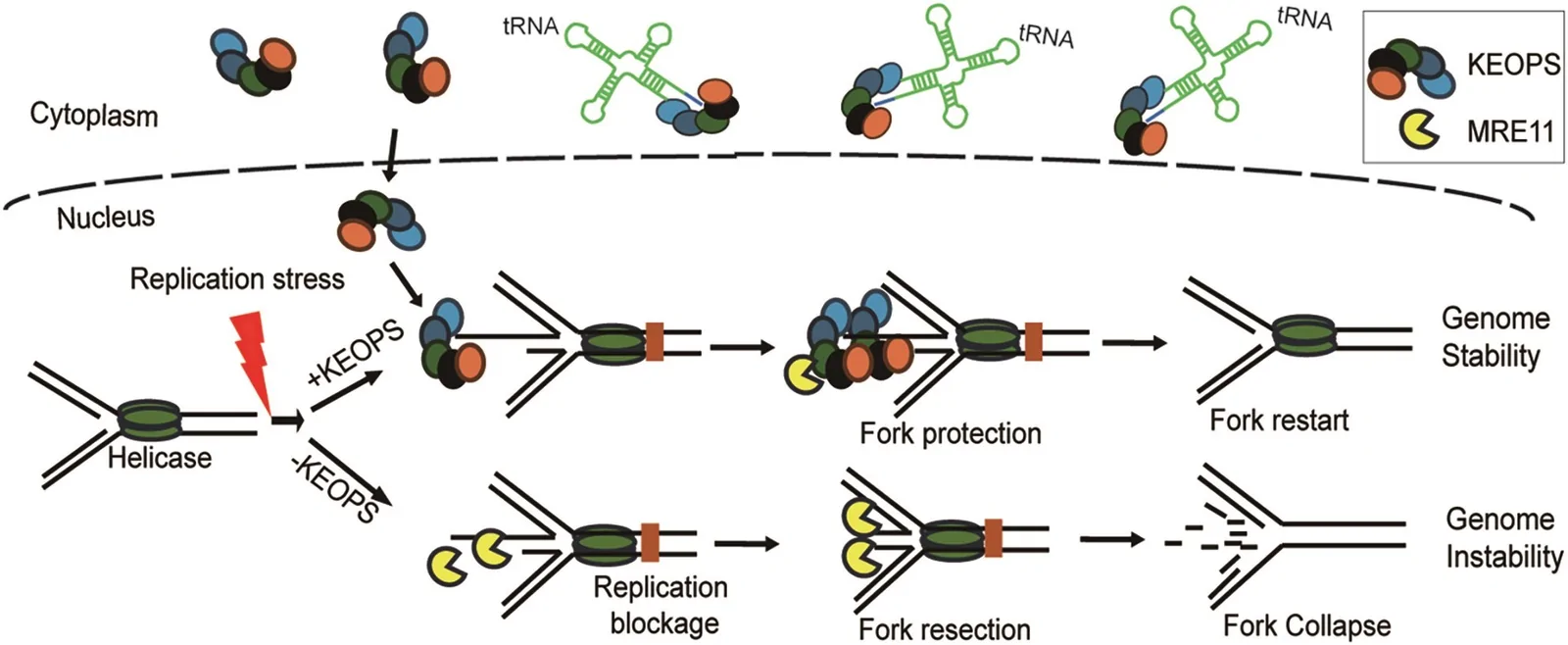

## Introduction

KEOPS complex, composed of OSGEP/Kea1, LAGE3/Pcc1, GON7, TPRKB/Cgi121, and TP53RK/Bud32 (human/yeast orthologs), is a highly conserved complex in eukaryotes and archaea [1]. Genetic analysis reveals that pathogenic mutations in human KEOPS (kinase, endopeptidase, and other proteins of small size) genes are the major causative factors for the development of a severe, rare disease, GAMOS, which leads to severe microcephaly and renal dysfunction, resulting in early childhood lethality [2]. Based on these studies on cellular pathways for KEOPS, knockdown of its subunits greatly suppresses cell proliferation, intervenes in protein translation, leads to endoplasmic reticulum stress, increases DNA damage repair (DDR) signaling, and therefore induces apoptosis [2]. Despite these insights, the molecular basis by which KEOPS dysfunction leads to GAMOS remains incompletely understood.

The KEOPS complex has been found to execute multiple cellular functions, mediating cellular fitness and organism development. Initially, the KEOPS complex was originally discovered through yeast genetic screens, and genome-wide search unveiled its mutations that rescued the growth of a *cdc13* mutant [3]. Therefore, the KEOPS complex has been demonstrated to be involved in telomere regulation and maintenance via Cdc13 protein [3, 4]. Subsequently, mutations in the KEOPS complex were found to be defective in general protein translation, which was then proven to be caused by a lack of tRNA modification [5]. Specifically, the KEOPS complex interacts with the 3′-CCA tail of tRNA to modify t6A37 [6, 7]. Beyond the capability of tRNA binding, the yeast KEOPS complex is also able to directly bind to chromatin DNA at DNA double strand break (DSB) sites, thereby regulating homologous recombination (HR)-mediated DNA damage repair (DDR) [8]. Recent evidence suggests that the functions of KEOPS in tRNA modification and chromosomal DNA regulation are independent of each other [4]. Until now, the roles of KEOPS in chromosomal DNA regulation have only been demonstrated in yeast, and whether these functions are also present in human cells remains to be determined.

LAGE3 (known as Pcc1 in yeast) occupies a central position within the subcomplex OSGEP-LAGE3-GON7 in the KEOPS complex. In podocytes, LAGE3 knockdown results in the strongest decrease in cell proliferation, and the highest rate of apoptosis compared to knockdown of OSGEP and GON7 [9]. The LAGE3 homolog Pcc1 in *Arabidopsis thaliana*, yeast, and archaea mediates the homodimerization of KEOPS, which is necessary for supporting cellular growth. Therefore, LAGE3 plays a core role in the function of the KEOPS complex. Biochemical studies have demonstrated that purified yeast KEOPS exhibits binding affinities for both single-stranded DNA (ssDNA) and double-stranded DNA (dsDNA). Structural analyses further reveal that among KEOPS subunits, only the Pcc1 possesses homology to the KH domain, a versatile nucleic acid-binding motif that can engage ssDNA, RNA, and RNA hairpins. Chromatin immunoprecipitation using Pcc1-TAP fusion proteins in yeast confirms its association with actively transcribed chromatin regions. Taken together, these findings suggest that LAGE3/Pcc1 may interact with chromosomal DNA and contribute to genome stability, including through HR-mediated DDR.

Previous reports have shown that KEOPS knockdown induces spontaneous genome instability in human cells, including increased γH2AX and p21 expression. Since DNA replication stress is the primary source of genome instability, we hypothesize that the KEOPS complex may participate in the cellular response to DNA replication stress and contribute to maintaining genome integrity. DNA replication stress is constantly induced by multiple endogenous and exogenous sources, leading to the formation of abnormal DNA structures that stall replication fork progression during S phase [10, 11]. If unresolved, stalled replication forks become susceptible to nucleolytic attack, resulting in fork collapse and subsequent genome instability [12]. Several protective proteins, such as RAD51, BRCA1/2, PARP1, and the CST complex, are recruited to stalled forks to stabilize and remodel them, thereby preventing the unscheduled degradation of nascent DNA strands by nucleases [13–17, 18]. Aberrant genome instability is closely linked to a spectrum of human diseases, including aging, cancer, and neurodegenerative disease. Consistently, LAGE3 expression has also been identified as a prognostic biomarker in several malignancies, including melanoma, colorectal cancer, hepatocellular carcinoma, and others [19–21, 22]. Given the KEOPS complex’s DNA-binding capacity and the KH domain–like structural features of LAGE3, we speculated that LAGE3 may associate with the replication forks and modulate the cellular response to replication stress [8].

In this study, we demonstrate that LAGE3 localizes to stalled forks under DNA replication stress. Loss of LAGE3 sensitizes cells to DNA-damage agents, indicating its protective role. TurboID-based proximity labeling reveals that MRE11 is a LAGE3-interacting partner following hydroxyurea (HU) treatment. DNA fiber assay confirms that LAGE3 protects stalled forks from MRE11-mediated degradation. Other KOPES components also localize to stalled forks, and TP53RK likewise mitigates MRE11-dependent fork resection under HU treatment. Moreover, we find that GAMOS-associated LAGE3 mutations disrupt KEOPS function, thereby exacerbating replication stress-induced genome instability. Together, our findings identify LAGE3 as a crucial fork protection factor that prevents MRE11-driven resection during replication stress, safeguarding genome stability. This study lays the groundwork for further mechanistic investigations into the role of KEOPS in DNA replication stress responses and the pathogenesis of GAMOS and related diseases.

## Materials and methods

### Cell culture

HeLa, HSkM, 293T, and U2OS cells were obtained from American Type Culture Collection (ATCC). Cells were cultured with DMEM media supplemented with 10% fetal bovine serum at 37°C containing 5% CO_2_.

### Antibodies

The following antibodies were used in this study. Anti-LAGE3 (WB 1:1000; PLA 1:200; Novus Biologicals, NBP2-32715); anti-GAPDH (WB 1: 60 000, Protech, 60004-1-Ig); anti-γH2AX (WB 1:5000, IF 1:3000, Active Motif, 39117); anti-H3 (WB 1:6000, Active Motif, 39763); anti-RPA32 (WB 1:5000, IF 1:2000, Protech, 10412-1-AP); anti-RPA32pS33 (WB 1: 2000, Abways, AY0326); anti-CHK1 (WB 1:2000, Protech, 25887-1-AP); anti-CHK1pS345 (WB 1:1000, Abclonal, A21009); anti-CldU (DNA fiber, 1:500, abcam, ab6326); anti-IdU (DNA fiber, 1:50, BD Biosciences, 347 580); anti-Flag (WB 1:3000; SIRF 1:2000; IF 1:2000, Protech, 66008-4-Ig); anti-biotin (SIRF,1:1000, Abclonal, A20684); anti-MRE11 (WB 1:2000, SIRF 1:1000, abclonal, A4222); HRP-streptavidin (WB 1:4000, Beyotime, A0305); Fluorescein (FITC)–conjugated Goat Anti-Rat IgG (H + L) (DNA fiber, 1:1000, Protech, SA00003-11); CoraLite594–conjugated Goat Anti-Mouse IgG (H + L) (DNA fiber, 1:1000, Proteintech, SA00013-3); CoraLite594-conjugated Goat Anti-Rabbit IgG (H + L) (IF, 1:2000, Proteintech, SA00013-4); Fluorescein (FITC)-conjugated Goat Anti-Mouse IgG (H + L) (IF, 1:2000, Proteintech, SA00003-1); HRP-conjugated Goat Anti-Rabbit IgG (H + L) (WB, 1:3000, Proteintech, SA00001-2); HRP-conjugated Goat Anti-Mouse IgG (H + L) (WB, 1:3000, Proteintech, SA00001-1).

### Immunofluorescence staining

Immunofluorescence staining was carried out as described previously [23]. Briefly, cells were cultured on the coverslips and washed with 1× PBS once, then fixed with 4% paraformaldehyde (PFA) in PBS for 15 min. The fixed cells were permeabilized with 0.15% Triton X-100 in PBS for 15 min, then washed with 1× PBS three times for 5 min. 10% BSA in PBS was used to block the samples for 1 h, incubated with primary antibodies overnight at 4°C. Samples were washed three times in PBS and then incubated with secondary antibodies for 1 h at room temperature. Finally, the samples on coverslips were washed three times and treated with cold ethanol series. Nuclei were stained with DAPI mounting medium. Images were obtained under ECHO microscope with 20× or 40× objective.

### Chromatin fraction isolation

Nuclear fractionation was carried out as described previously [24]. Briefly, cells (1 × 10^6^) were resuspended in buffer A (10 mM HEPES, pH = 7.9, 1.5 mM MgCl_2_, 10 mM KCl, 1 mM DTT, protease inhibitor cocktail) and incubated on ice for 5 min. Then nuclei were collected in the pellet fraction by centrifuging at 1300 g for 4 min at 4°C. The nuclei were washed once with buffer A, followed by the addition of buffer B (3 mM EDTA, 0.2 mM EGTA, 1 mM DTT, protease inhibitor cocktail) for 10 min to lyse the nuclei. Centrifugation at 1700 × *g*, 4 min, 4°C was used to collect the insoluble chromatin fraction in the pellet and the nuclear soluble fraction (NS) in the supernatant. The pellet was washed once with buffer B and centrifuged again. The final enriched chromatin pellet (CP) was dissolved in Laemmli buffer and sonicated.

### Auxin-induced degradation of LAGE3

To rapidly degrade LAGE3 protein, HeLa cells were stably transfected to express OsTIR1 (F74G) and an siRNA-resistant LAGE3-AID fusion protein. Subsequently, siLAGE3 was transfected into these engineered HeLa cells to specifically deplete endogenous LAGE3 while preserving the siRNA-resistant LAGE3-AID. After 48 h, endogenous LAGE3 (but not the siRNA-resistant LAGE3-AID) was efficiently knocked down. Finally, treatment with 0.25 μM 5-ph-IAA for 6 h induced rapid degradation of the AID-tagged LAGE3 protein.

### TurboID-based biotinylated protein enrichment

To identify the proximal proteins of LAGE3 in response to DNA replication stress, we established HEK293T cells stably expressing LAGE3-TurboID, using GFP-TurboID as a control. All cells were either directly labeled with 500 μM biotin or first treated with 4 mM HU for 5 h prior to biotin labeling. The biotin labeling was performed at 37°C with 5% CO_2_. Following labeling, the cells were washed five times with cold PBS and subsequently lysed with cold RIPA buffer (50 mM Tris–HCl pH 8.0, 150 mM NaCl, 0.1% SDS, 0.5% sodium deoxycholate, 1% Triton X-100, and protease inhibitor cocktail). The cell lysates were centrifuged at 13 000 × *g* for 10 min at 4°C. The resulting supernatant was incubated with magnetic streptavidin beads for 5 h at 4°C. The beads were then subjected to a series of washes, twice with RIPA buffer, once with 1 M KCl, once with 0.1 M Na_2_CO_3_, once with 2 M urea in 10 mM Tris–HCl (pH 8.0), and finally twice more with RIPA buffer. Biotinylated proteins bound to the beads were identified by mass spectrometry analysis.

### On-bead trypsin digestion of biotinylated proteins

Prior to mass spectrometry analysis, streptavidin bead-bound biotinylated proteins were subjected to on-bead trypsin digestion as previously described [25]. Briefly, the beads were sequentially washed with 50 mM Tris–HCl buffer (pH 7.5) and 2 M urea/50 mM Tris–HCl (pH 7.5) buffer. On-bead digestion was then initiated by incubating the beads in 80 μl digesting buffer (2 M urea/50 mM Tris–HCl, 1 mM DTT, 0.4 μg trypsin) for 1 h at 25°C with shaking. The supernatant was collected, and beads were washed twice with 60 μl of 2 M urea/50 mM Tris–HCl buffer (pH 7.5). All washes were combined with the initial digest supernatant. The combined eluate was then reduced with 4 mM DTT for 30 min at 25°C with shaking, followed by alkylation with 10 mM iodoacetamide for 45 min in the dark at 25°C with shaking. To ensure complete digestion, an additional 0.5 μg of trypsin was added, and the reaction was allowed to proceed overnight at 25°C with shaking. Finally, the digest was acidified to pH < 3 by adding formic acid to a final concentration of ~1%.

### Liquid chromatography and mass spectrometry analysis

The peptide sample was first loaded onto the C18-reversed-phase analytical column (Thermo Scientific, Acclaim PepMap RSLC 50 μm × 15 cm, nano viper, P/N 164943) in buffer A (0.1% formic acid in HPLC-grade water) and separated with a linear gradient of buffer B (80% acetonitrile and 0.1% formic acid) with a flow rate at 300 nl/min. The linear chromatographic gradient was achieved with linear increase of buffer B percentage, which was set up as follows: 6% buffer B for 5 min, 6%–28% buffer B for 40 min, 28%–38% buffer B for 5 min, 38%–100% buffer B for 5 min, hold in 100% buffer B for 5 min. After that, the peptide entered into the Q Exactive mass spectrometer (Thermo Fisher Scientific, MA, USA). The MS analysis was set for 60 min in a positive ion mode. MS data were acquired using a data-dependent Top10 method dynamically choosing the most abundant precursor ions from the full scan (350–1800 m/z) for higher-energy collision dissociation (HCD) fragmentation. Full scans were acquired at a resolution of 70 000 at m/z 200 with an automatic gain control (AGC) target of 3e6 and a maxIT of 50 ms. MS2 scans were acquired at a resolution of 17 500 for HCD spectra at m/z 200 with an AGC target of 2e^5^ and a maxIT of 45 ms. The scan range was 200–2000 m/z and the isolation width was 2 m/z. Only ions with a charge state between 2 and 7 and a minimum intensity of 2e^3^ were selected for fragmentation. Dynamic exclusion for selected ions was 30 s. The microscan is 1. The normalized collision energy was 27 eV.

### MS data analysis

MS data were processed with MaxQuant (version 1.6.17.0) and searched against the UniProt Homo sapiens database (release 2024_05, 20 434 entries) using the built-in Andromeda search engine. The search settings specified trypsin/P as the protease and allowed up to four missed cleavages. Carbamidomethylation of cysteine was set as a fixed modification, while protein N-terminal acetylation and methionine oxidation were set as variable modifications. The false discovery rate (FDR) was controlled at 1% at both the peptide and protein levels.

To screen the proteins specifically proximal to LAGE3, we selected the proteins based on a higher LFQ intensity in the LAGE3 samples compared to the GFP control in both the HU-treated and untreated groups. Subsequently, proteins with higher LFQ intensity in LAGE3-TurboID with HU treatment compared to that without HU treatment were considered proximal proteins under DNA replication stress (i.e. LFQ Intensity_LAGE3-TurboID+HU_/LFQ Intensity_LAGE3-TurboID no HU_ > 1). Finally, these proximal proteins to LAGE3 in response to DNA replication stress were used for GO Biological Processes analysis with Metascape database [26]. All the raw data have been deposited in ProteomeXchange (ID: PXD066518).

### PLA assay

PLA assay was conducted as described previously [27]. Briefly, cells were grown on the coverslips and then fixed directly with 4% PFA in PBS for 15 min, then permeabilized in 0.15% Triton X-100 in PBS for 15 min, and washed three times for 5 min each with PBS. Cells were blocked with 10% BSA in PBS at 37°C for 1 h and then incubated with indicated primary antibodies overnight at 4°C. Samples were washed with wash buffer A (0.01 M Tris, 0.15 M NaCl, and 0.05% Tween-20, pH 7.4) three times for 5 min each, and incubated with Duolink *in situ* PLA probe anti-mouse plus and anti-rabbit minus for 1 h at 37°C. Then cells were washed again with wash buffer A three times for 5 min each, incubated with Duolink ligation mix at 37°C for 30 min. The slides were washed with buffer A twice for 2 min each, incubated with Duolink amplification mix at 37°C for 100 min, washed with wash buffer B (0.2 M Tris and 0.1 M NaCl) three times for 10 min each, and washed once with 0.01× diluted wash buffer B for 1 min. Finally, the samples were stained with DAPI, and the images were obtained under ECHO microscope with a 40× objective.

### SIRF assay

SIRF assay was performed as described previously [23]. Briefly, cells were cultured on the coverslips and labeled with EdU for 8 min. To induce replication fork stalling, EdU was removed by washing with PBS and then treated with 4 mM HU for 3 h. Subsequently, cells were pre-permeabilized with 0.25% Triton X-100 for 3 min prior to being fixed with 4% PFA for 15 min at room temperature, then washed with PBS twice. After permeabilization with 0.25% Triton X-100 for 15 min at room temperature, the cells were washed three times for 5 min each with PBS. Cells were incubated with click reaction buffer (2 mM copper sulfate, 10 µM biotin-azide, and 100 mM sodium ascorbate) at 37°C for 1 h, then washed three times for 5 min with PBS and then blocked with blocking buffer. PLA procedures with anti-biotin and anti-FLAG antibodies were then performed in PLA assay (see above).

### DNA fiber analysis

DNA fiber assay was performed as described previously [23]. Briefly, cells were first labeled with 25 μM CldU for 30 min, then followed by 250 μM IdU for another 30 min. To induce DNA replication stress, cells were treated with 4 mM HU for 5 h, and 50 μM mirin was concomitantly added with HU as indicated. The cells were collected and resuspended in PBS, lysed in lysis buffer (200 mM Tris–HCl pH 7.4, 50 mM EDTA, 0.5% SDS); then DNA fibers were stretched on the slides. Subsequently, the stretched DNA fibers were fixed in methanol:acetic acid (3:1) and then denatured with 2.5 M HCl for 80 min. The slides were washed with PBS and blocked with 5% BSA for 30 min. The CldU and IdU labeled nascent DNA were immunostained with anti-CldU and anti-IdU antibodies, then incubated with anti-rat Alexa Fluor 488 and anti-mouse Alexa Fluor 568 secondary antibodies. Coverslips were washed and mounted with mounting medium. Images were acquired with ECHO microscope at 40× magnification, and the length of DNA fiber was measured using the ImageJ software.

### Protein expression and purification

#### hKEOPS complex and its individual components

The polycistronic plasmid PET24a-hKEOPS (expressing OSGEP, TP53RK, TPRKB, LAGE3, and GON7-His_6_) was synthesized by BGI Liuhe Co. Ltd, in which the coding codons in expression genes were optimized according to the codon usage bias of *Escherichia coli*. Individual genes were cloned and inserted into pET28a by a Seamless Cloning Kit (Beyotime, Shanghai, China). The subcomplex of KEOPS complex *OSGEP-LAGE3-GON7* and *TP53RK-TPRKB* genes were cloned from PET24a-hKEOPS and inserted into pET24a vector. Gene expression vectors were expressed in *E. coli* BL21 (DE3) cells. Protein purification was initially performed according to a previously described method [23].

Human MRE11 protein was purchased from Biodragon company (Cat#BA54625), which was purified from HEK293T cells.

### Preparation of the DNA substrates

Fluorescence-labeled DNA substrates were prepared by annealing the synthetic oligonucleotides described below [23]. For ds-ssDNA oligonucleotides, 59 nt ssDNA with the modified phosphorothioate bond (labeled with an asterisk) at both ends to prevent the non-specific nucleases digestion. All the oligonucleotides were shown as follows:

Oligo 1: 5′- A*C*G*C*T*CCGAATTCTACCAGTGCCTTGCTAGGACATCTTTGCCCACCTGCAGGTTC*A*C*C*C* -3′;

Oligo 2: 5′-Cy5.5-GGGTGAACCTGCAGGTGGGCAAAGA −3′;

Oligo 3: 5′-Cy5.5-AGGGACATTGTGCTGTGCGGTGAGGTTTGTCTTTGCCCACCTGCAGGTTC−3′

Oligo 4: 5′-GAACCTGCAGGTGGGCAAAGACAAACCTCACCGCACAGCACAATGTCCCT-3′

For ds-ssDNA and dsDNA oligonucleotides, equal amounts of oligonucleotides (oligo 1 + 2, oligo 3 + 4) were mixed in the annealing buffer (50 mM Tris pH 7.5, 10 mM MgCl_2_, 100 mM NaCl, and 1 mM DTT) and heated at 80°C for 3 min. The mixed reaction was subsequently transferred to 65°C for 30 min and cooled down slowly to room temperature. The annealed substrate was purified from a 10% native polyacrylamide gel by electro-elution and filter-dialyzed in an Amicon ultra-4 concentrator at 4°C into TE buffer (10 mM Tris–HCl, pH 8.0, and 0.5 mM EDTA). For ssDNA, Oligo 3 was directly used.

### Electrophoretic mobility shift assay

Electrophoretic mobility shift assay (EMSA) was performed as described previously [23]. Briefly, the Cy5.5-labeled 5′ overhang DNA substrate (80 nM) was incubated with specified concentrations of either KEOPS or its individual component in 10 μl reaction buffer (35 mM Tris–HCl pH 7.5, 1 mM DTT, 100 ng/μl BSA, 50 mM KCl) at 37°C for 5 min. Reaction products were then electrophoresed on 4% EMSA PAGE gel in 0.5× TBE buffer at 100 V for 40 min. Fluorescent detection was performed using Odyssey DLx Imaging System.

### MRE11 nuclease assay

MRE11 degradation assay was performed as described previously [23]. Briefly, the Cy5.5-labeled 5′ overhang DNA substrate (80 nM) was incubated with specified concentrations of KEOPS complex in 10 μl reaction buffer (35 mM Tris–HCl pH 7.5, 1 mM DTT, 100 ng/μl BSA, 50 mM KCl, 2.5 mM MgCl₂, 1 mM ATP, 1 mM MnCl₂) at 37°C for 5 min. Purified human MRE11 (200 nM) was then added and reactions continued at 37°C for 20 or 40 min. Reactions were terminated with 2.5 μl stop buffer (50 mM EDTA, 0.4% SDS, 3.2 mg/ml proteinase K) at 37°C for 15 min. For analysis, samples were mixed with equal volume of 2× denaturing dye (95% formamide, 0.1% Orange G, 10 mM Tris–HCl pH 7.5, 1 mM EDTA, 12% Ficoll PM400), heat-denatured at 95°C for 10 min, and resolved on 27% denaturing TBE-urea-PAGE (7 M urea) in 1× TBE buffer at 300 V for 40 min at 55°C. Fluorescent products were visualized with the Odyssey DLx Imaging System and quantified with ImageJ software.

## Results

### LAGE3 deficiency drives DNA damage accumulation and genome instability

**Figure 1.**
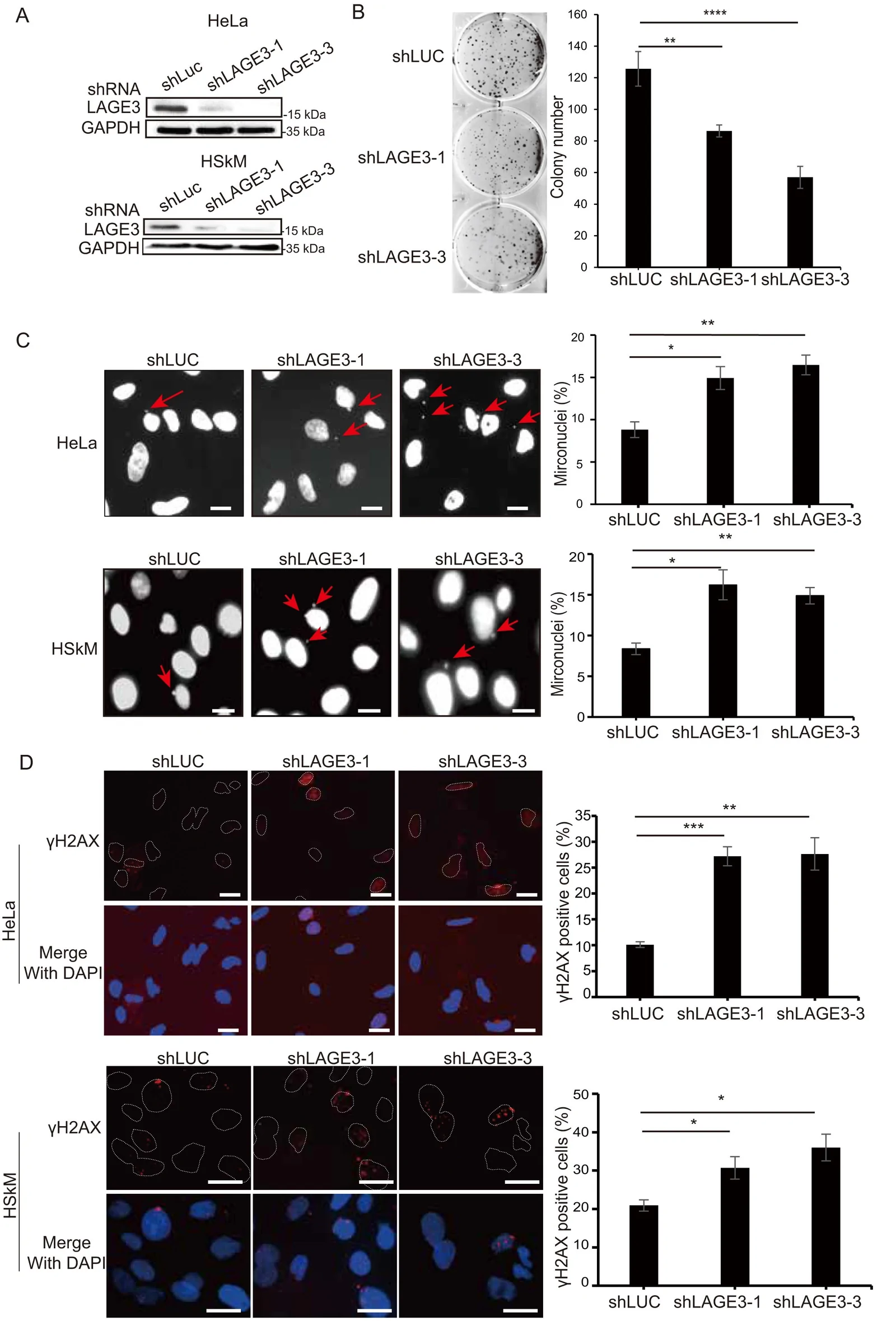
LAGE3 deficiency drives DNA damage accumulation and genome instability. (**A**) Western blot showing LAGE3 knock-down in HeLa and HSkM cells. Two shRNAs targeting LAGE3 were used and shLUC was used as the control. (**B**) LAGE3 deficiency inhibits cell proliferation via colony formation analysis. Cell colonies were quantified from three independent experiments. *P* values were calculated by one-way ANOVA analysis, **P < .01, ^****^P < .0001. Error bars: SEM. (**C**) Micronuclei were stained with DPAI in LAGE3-depleted HeLa and HSkM cells. The percentage of cells with micronuclei was quantified from three independent experiments. *P* values were calculated by one-way ANOVA analysis, *P < .05, **P < .01. Error bars: SEM. Scale bar: 10 μm. (**D**) γH2AX immunofluorescence staining was performed in LAGE3-deficient HeLa and HSkM cells. The nucleus with >5 foci were considered as γH2AX-positive cell. The percentage of γH2AX positive cells was quantified from three independent experiments. *P* values were calculated by one-way ANOVA analysis, *P < .05, **P < .01, ***P < .001. Error bars: SEM. Scale bar: 10 μm.

Human KEOPS complex and its orthologs in other species have been implicated in genome instability beyond their established role in t6A tRNA modification [2]. Since the deficiency of KEOPS complex induces spontaneous genome instability and growth inhibition in both normal and cancer cells, we hypothesized that KEOPS complex has additional DNA protective functions beyond HR and telomere protection [2, 8]. LAGE3, a key component of KEOPS, mediates KEOPS dimerization, a function critical to its overall activity [7, 28, 29]. To investigate the role of LAGE3 in genome maintenance, we knocked down LAGE3 in both HeLa cancer cells and non-transformed HSkM cells using two independent shRNA sequences, shLAGE3-1 and shLAGE3-3 (Fig. 1A). Cell proliferation assays, including CCK-8 and colony formation, showed that LAGE3 depletion markedly inhibited proliferation, consistent with its reported role in tumorigenesis (Fig. 1B and Supplementary Fig. S1A). Meanwhile, Calcein-AM/PI staining further confirmed a significant increase in cell death upon LAGE3 knockdown (Supplementary Fig. S1B).

To assess whether LAGE3 deficiency induces spontaneous genomic instability, we examined micronuclei (MN) formation, a widely accepted marker of chromosomal damage. Both HeLa and HSkM cells showed significantly increased MN frequency upon LAGE3 knockdown (Fig. 1C). Moreover, LAGE3-deficient cells exhibited elevated levels of γH2AX, a marker of DNA damage, along with G2/M cell cycle arrest as detected by flow cytometry (Fig. 1D and Supplementary Fig. S1C and D). These findings suggest that LAGE3 plays a crucial role in maintaining genome stability by inhibiting endogenous DNA damage and preventing the accumulation of chromosomal lesions.

### LAGE3 acts as a genomic guardian to counteract DNA replication stress and maintains genome stability

**Figure 2.**
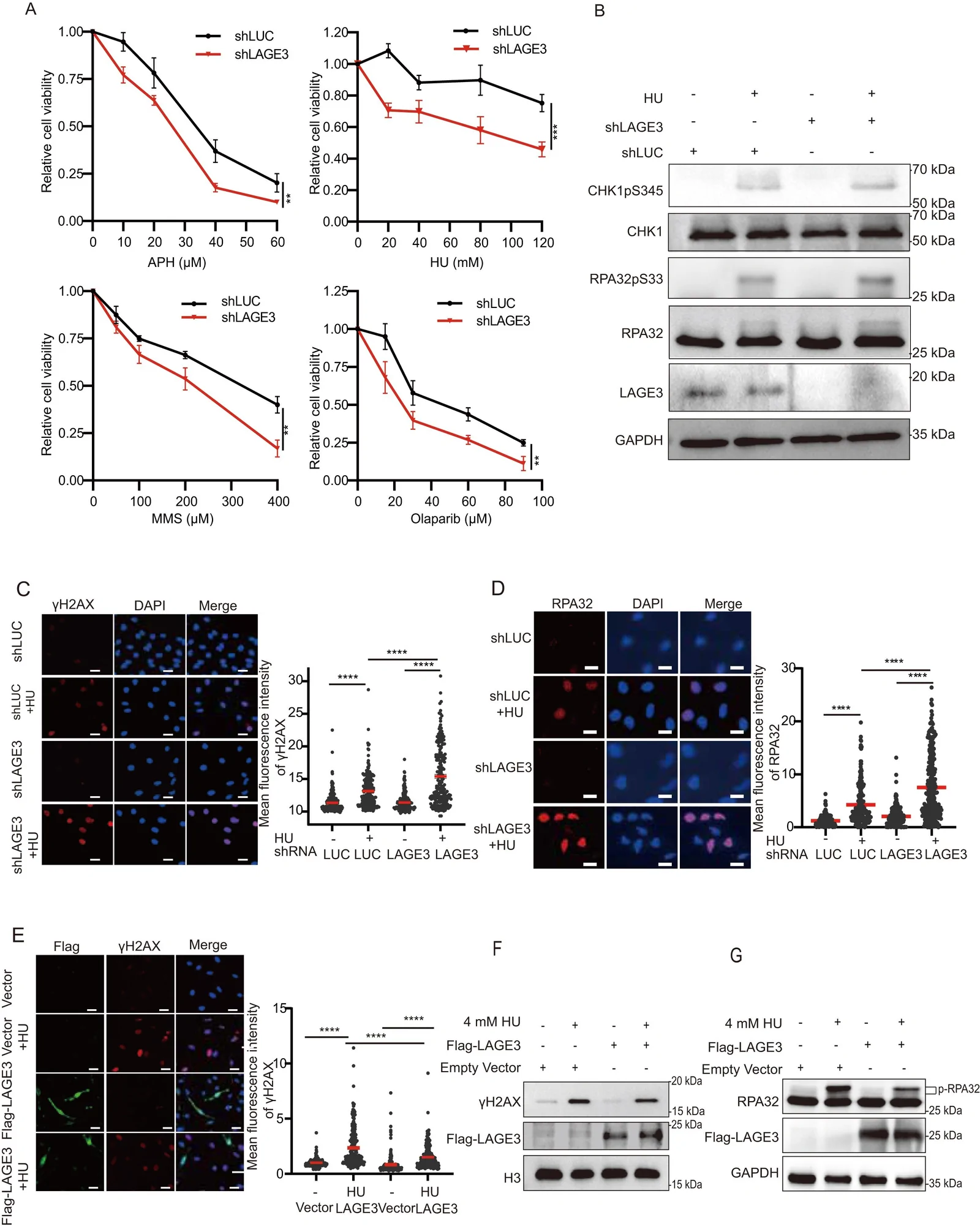
LAGE3 modulates cellular response to DNA replication stress. (**A**) CCK8 assay showing cell sensitivity to different replication stressors with indicated concentrations for LAGE3-deficient cells. The data were collected from three independent experiments. Statistical analysis was performed with two-way ANOVA, **P < .01, ***P < .001. Error bars: SEM. (**B**) LAGE3 deficiency increases CHK1pS345 and RPA32pS33 expression under DNA replication stress. (**C**) LAGE3 deficiency increases γH2AX expression under DNA replication stress by immunofluorescence staining. The fluorescence intensity was obtained from three independent experiments. Statistical analysis was performed with one-way ANOVA, ^****^P < .0001. Error bars: SEM. Scale bar: 20 μm. (**D**) LAGE3 deficiency increases the nuclear RPA32 expression in response to DNA replication stress. The fluorescence intensity was obtained from three independent experiments. Statistical analysis was performed with one-way ANOVA, ^****^P < .0001. Error bars: SEM. Scale bar: 20 μm. (**E**) LAGE3 overexpression decreases γH2AX expression in response to DNA replication stress. The fluorescence intensity was obtained from three independent experiments. Statistical analysis was performed with one-way ANOVA, ^****^P < .0001. Error bars: SEM. Scale bar: 20 μm. (**F**) Western blot showing a decrease of γH2AX expression in stably expressed LAGE3 cells in response to DNA replication stress. (**G**) LAGE3 overexpression decreases RPA32 phosphorylation in response to DNA replication stress.

Previous studies have evidenced that the DNA lesions caused by replication stress are the major source for spontaneous genome instability, which prompted us to investigate whether LAGE3 modulates the progression of DNA replication stress response [30]. To address this question, we first used the CCK-8 assay to measure the cell viability following treatment with four different DNA replication stress-inducing agents, including aphidicolin (APH), hydroxyurea (HU), methyl methanesulfonate (MMS), and Olaparib, which target different DNA replication processes. LAGE3-deficient cells exhibited heightened sensitivity to all treatments, highlighting a protective role for LAGE3 in the replication stress response and genome maintenance (Fig. 2A).

The ATR signaling pathway is the central regulator of cellular responses to DNA replication stress [31]. Therefore, we examined the phosphorylation of the key ATR effectors, RPA32pS33 and CHK1pS345. Western blot analysis exhibits enhanced phosphorylation of both substrates in LAGE3-deficient cells with HU treatment, implying that LAGE3 can mitigate DNA lesions under DNA replication stress (Fig. 2B and Supplementary Fig. S2A and B). Consistently, immunofluorescence staining also revealed a significant increase in γH2AX foci formation following HU treatment in LAGE3-deficient cells, implying the exacerbated DNA lesions (Fig. 2C). The aberrant accumulation of ssDNA is considered the first outcome of fork stalling and an important hallmark of DNA replication stress-induced genome instability. And RPA (replication protein A) complex is the predominant ssDNA-binding factor in eukaryotes to stabilize the excessive ssDNA stretches. We observed a significant increase in nuclear RPA32 foci formation in LAGE3-depleted HeLa cells under HU treatment, consistent with the observation that LAGE3 deficiency impairs the stability of stalled forks (Fig. 2D).

To further validate the protective role of LAGE3 in genome stability, we transiently overexpressed LAGE3 in HeLa cells. We found that it significantly reduced γH2AX foci formation by immunofluorescence (Fig. 2E). Consistent with this, western blot analysis demonstrated that stable ectopic LAGE3 expression also lowered γH2AX levels (Fig. 2F). Moreover, phosphorylation of RPA32 was downregulated in LAGE3-overexpressing cells (Fig. 2G), further supporting the conclusion that LAGE3 alleviates replication stress-associated DNA damage.

### LAGE3 is recruited to the chromatin and localizes on stalled forks under replication stress

**Figure 3.**
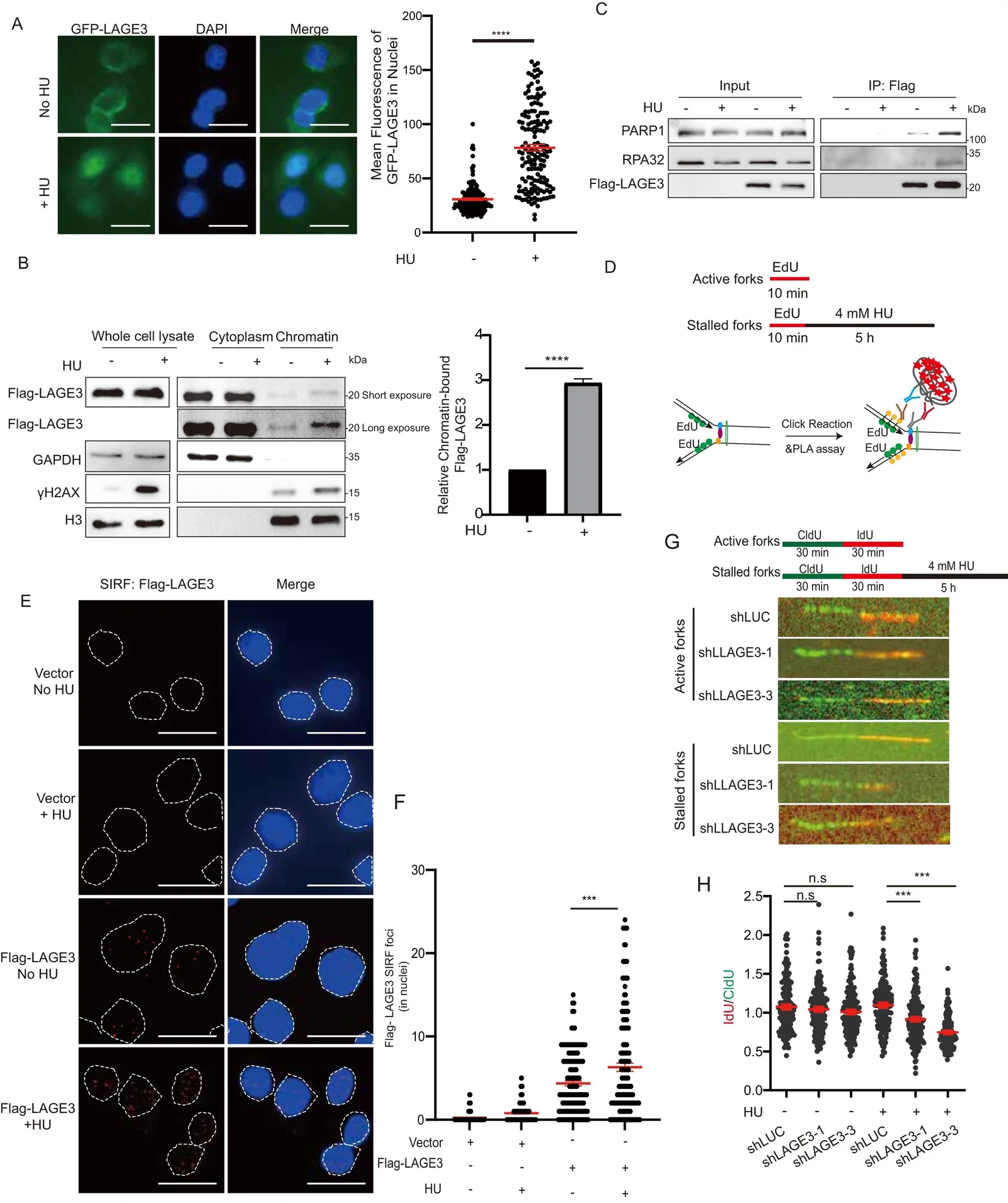
LAGE3 localizes at stalled forks. (**A**) GFP-LAGE3 in HeLa cells exhibits replication stress-induced nuclear accumulation. Relative fluorescent intensity of GFP-LAGE3 is plotted. Statistical analysis was performed with unpaired Student’s t test, ^****^P < .0001. Error bars: SEM. Scale bar: 20 μm. (**B**) Flag-LAGE3 were recruited to chromatin in response to DNA replication stress. Chromatin-associated proteins were isolated, and the corresponding proteins were analyzed by western blot. GAPDH and H3 were used as the markers for cytoplasmic and chromatin fractions, respectively, and γH2AX expression was considered the marker for HU-induced DNA replication stress. (**C**) CoIP assay indicates the interaction of Flag-LAGE3 with PARP1 and RPA32 under replication stress in HEK293T cells. (**D**) Schematic of the pulse EdU-labeling procedure with and without HU treatment (upper panel) and SIRF assay (lower panel). (**E**) LAGE3 was detected at both the active and stalled forks by SIRF assay. Representative SIRF images of Flag-LAGE3 were shown. Scale bar: 20 μm. (**F**) Flag-LAGE3 SIRF foci were quantified. Mean value was labeled. At least 100 cells were quantified in each sample. Data were collected from two biological repeats, and statistical analysis was performed with one-way ANOVA, ^****^P < .0001. Error bars: SEM. (**G**) Schematic of the DNA fiber assay with pulse-labeling CldU and IdU, and the representative fiber image in each group. (**H**) DNA fiber analysis of the stability of replication forks with and without HU treatment in LAGE3-deficient cells. The ratio of IdU/CldU was used to evaluate the stability of nascent stranded DNA. Data were collected from two biological repeats and statistical analysis was performed with one-way ANOVA, ***P < .001, Error bars: SEM.

Protein subcellular localization is fundamental to its biological function. We established HeLa cell lines stably expressing either GFP- or Flag-tagged LAGE3. Both cell lines showed significant nuclear accumulation of LAGE3 following HU treatment, demonstrating that DNA replication stress induces nuclear enrichment of LAGE3 (Fig. 3A and Supplementary Fig. S3A). Subsequently, using a previously established chromatin fractionation protocol, we isolated chromatin-bound proteins from HeLa cells stably expressing Flag-tagged LAGE3 [24]. Histone H3 and GAPDH were used as the markers for nuclear and cytoplasmic fractions, respectively, while γH2AX served as a readout for HU-induced replication stress. Although a substantial portion of LAGE3 remained cytoplasmic, its chromatin-bound fraction markedly increased upon HU treatment, suggesting stress-induced recruitment to chromatin (Fig. 3B).

Next, we transfected Flag-LAGE3 into HEK293T cells and then utilized Co-IP to analyze the interaction of LAGE3 with replication stress-associated proteins. RPA32 and PARP1 were demonstrated to interact with LAGE3 in response to DNA replication stress (Fig. 3C). Previous studies revealed that both the RPA complex and PARP1 directly participate in stalled fork processing, which prompted us to hypothesize that LAGE3 is also involved in the progression of stalled forks.

To determine whether LAGE3 directly binds to stalled forks, we performed the SIRF (*in situ* analysis of protein interactions at DNA replication forks) assay, which allows for single-cell visualization and quantification of fork-associated proteins (Fig. 3D) [32]. LAGE3 SIRF signals were significantly enriched on the stalled forks upon HU treatment, implying a direct role of LAGE3 in the processing of the stalled forks (Fig. 3E and F). Next, we performed the DNA fiber assay to investigate the role of LAGE3 in maintaining the stability of replication forks. Through pulse-labeling CldU and IdU sequentially and 4 mM HU treatment to induce DNA replication stress as shown in Fig. 3G, the nascent strand DNA was evaluated by immunofluorescence staining. Knockdown of LAGE3 leads to degradation of nascent-strand DNA under replication stress, indicating the instability of stalled forks (Fig. 3G and H). While the t6A tRNA modification activity of KEOPS would affect the general protein translation, it is challenging to determine either indirectly t6A tRNA modification activity or directly the chromatin binding ability that protects the stalled fork stability. Theoretically, short-term depletion of KEOPS does not affect the temporary protein translation process; however, it will lead to the loss of chromatin binding. Thus, we established an AID2-based rapid protein degradation system targeting LAGE3-AID and observed that the majority of LAGE3 proteins were degraded within 5 hours (see the “Materials and methods” section) (Supplementary Fig. S3B and C). Subsequent DNA fiber assay revealed that transient LAGE3 depletion also destabilized the stalled replication forks to a similar extent as those caused by long-term depletion with shRNA (Supplementary Fig. S3D and Fig. 3H).

Together, these findings demonstrate that LAGE3 is recruited to chromatin and localizes at stalled replication forks under replication stress, where it protects nascent DNA from aberrant degradation, thereby preserving genome integrity.

### LAGE3 protects the stalled replication forks by limiting MRE11 endonuclease activity

Our findings thus far support the conclusion that LAGE3 plays a critical role in protecting stalled forks to maintain genome stability. Therefore, to further investigate the underlying molecular mechanism, we performed a TurboID-based proximity labeling assay to identify the LAGE3 proximal proteome under replication stress. The TurboID-Flag-tagged LAGE3 was stably expressed in 293T via lentivirus transduction. Cells were labeled with 500 μM biotin for the indicated time and then stopped labeling by placing the cells on ice as described previously [25]. Immunobloting assay confirmed that a 40-minute labeling period was sufficient to achieve robust protein biotinylation (Supplementary Fig. S4A).

**Figure 4.**
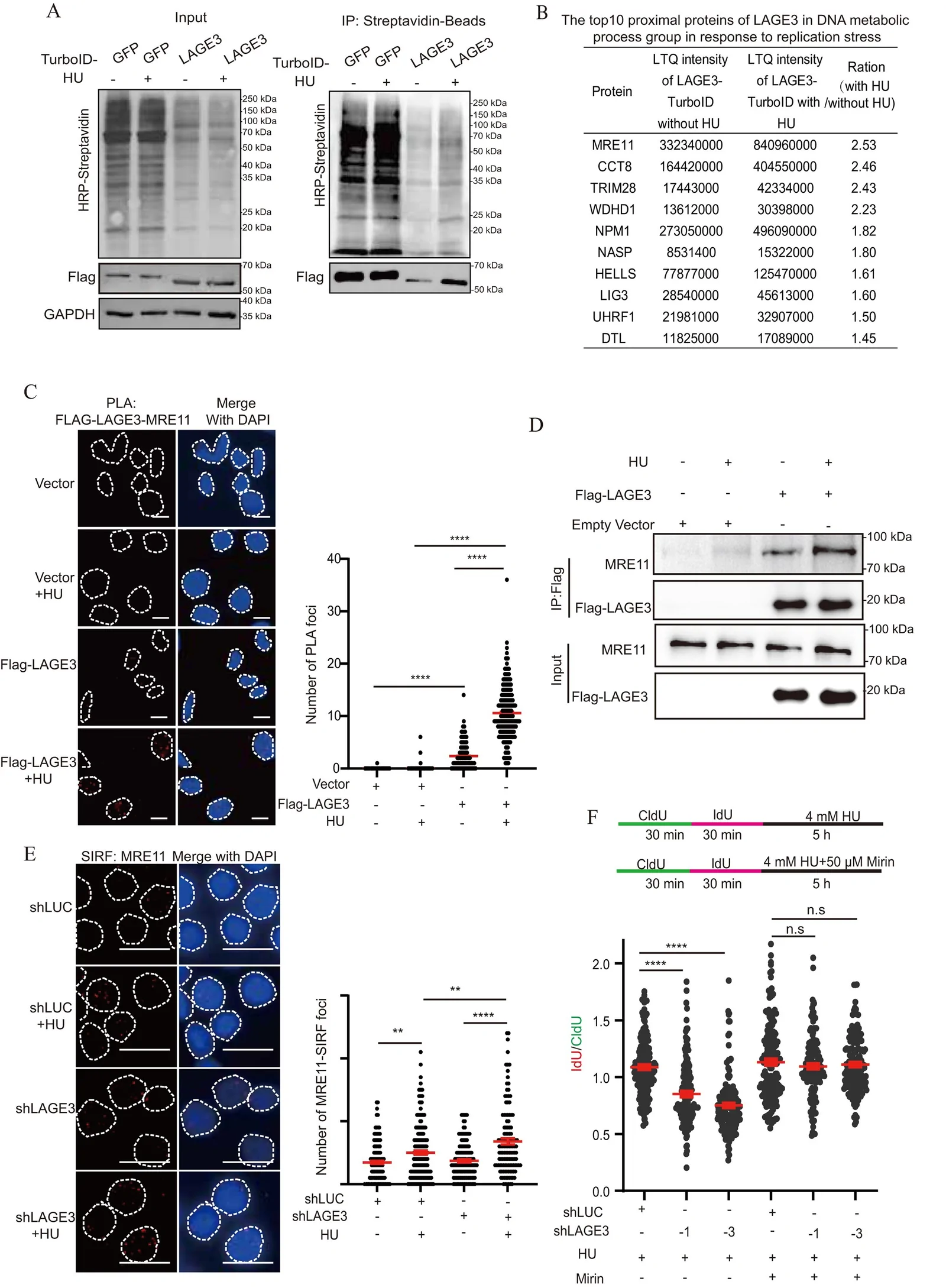
LAGE3 protects stalled forks from MRE11 nuclease degradation. (**A**) Western blot showing the biotinylated LAGE3 proximal proteins with and without HU treatment. The biotinylated proteins were enriched by magnetic streptavidin beads. (**B**) The list of top 10 proximal proteins of LAGE3 in DNA metabolic process group in response to DNA replication stress. (**C**) Representative images and quantification of PLA foci between Flag-LAGE3 and endogenous MRE11 with and without HU treatment. Each dot indicates the PLA foci number in one cell. Scale bars: 10 μm. Data were collected from two biological repeats and statistical analysis was performed with one-way ANOVA, ^****^P < .0001, Error bars: SEM. (**D**) CoIP analysis of the interaction between FLAG-LAGE3 and endogenous MRE11 with and without HU treatment. (**E**) SIRF analysis of the localization of MRE11 at active and stalled forks in LAGE3-deficient cells. The number of MRE11 SIRF foci in nuclei was quantified. Mean value was labeled. At least 100 cells were quantified in each sample. Data were collected from two biological repeats and statistical analysis was performed with one-way ANOVA, ^****^P < .0001. Error bars: SEM. (**F**) DNA fiber analysis of the stability of stalled replication forks with and without MRE11 inhibitor mirin treatment in LAGE3-deficient cells. The ratio of IdU/CldU was used to evaluate the stability of nascent stranded DNA. Data were collected from two biological repeats and statistical analysis was performed with one-way ANOVA, ^****^P < .0001, Error bars: SEM.

To identify proteins interacting with LAGE3 under replication stress conditions, we used TurboID-GFP as a negetive control and treated 293T cells expressing TurboID-FLAG-LAGE3 with and without 2 mM HU for 5 h, followed by 500 μM biotin labeling for 40 min. Biotinylated LAGE3-proximal proteins were isolated using magnetic streptavidin beads and analyzed by western blotting (Fig. 4A). Mass spectrometry identified 437 proteins specifically enriched in LAGE3-proximal samples (Table 1). Gene Ontology (GO) enrichment analysis using the Metascape database revealed a significant enrichment in the “DNA metabolic process” category upon HU treatment (Supplementary Fig. S4B). Within this group, quantitative analysis of protein levels by LTQ intensity identified MRE11 as the most upregulated protein within this group in response to DNA replication stress, with the top 10 proteins listed in Fig. 4B.

**Table 1.** The proximal proteins of LAGE3 with TurboID

Gene name	LFQ intensity GFP	LFQ intensity GFP with HU	LFQ intensity LAGE3	LFQ intensity LAGE3 with HU
PPP2R5D	0	309 840 000	35 761 000	347 360 000
CCDC28A	0	0	3 965 700	19 701 000
POP7	19 332 000	0	39 722 000	189 980 000
CNDP2	0	9 824 300	9 861 700	40 894 000
ZYX	17 905 000	0	29 514 000	120 990 000
MCCC1	8 946 500	8 743 900	34 848 000	141 330 000
CEP170	8 588 000	19 284 000	18 867 000	75 393 000
CIAPIN1	4 337 100	0	7 812 900	29 476 000
CD2AP	0	8 099 700	8 398 000	28 228 000
SYNRG	8 772 900	4 565 200	13 280 000	43 362 000
SLC4A1AP	0	6 900 800	7 277 300	21 022 000
NUDC	14 691 000	15 308 000	26 318 000	73 923 000
MAP1B	0	0	10 753 000	27 296 000
MRE11A	99 013 000	119 370 000	332 340 000	840 960 000
CHMP4B	12 175 000	14 708 000	16 337 000	41 291 000
ANKRD17	3 002 200	0	4 050 000	10 219 000
TTC4	10 848 000	9 819 700	13 321 000	33 515 000
CCT8	87 434 000	89 140 000	164 420 000	404 550 000
SYAP1	0	0	8 565 500	20 424 000
TMOD3	0	7 304 600	6 741 000	15 932 000
PPP1R12A	0	0	4 894 500	11 176 000
TJP1	8 576 000	11 277 000	14 031 000	31 754 000
RPAP3	18 223 000	15 228 000	65 114 000	144 200 000
PAK2	23 819 000	21 330 000	63 734 000	136 170 000
PDLIM7	2 920 100	2 687 100	4 809 600	10 266 000
ENSA	18 341 000	20 612 000	22 592 000	47 726 000
LAGE3	0	0	52 634 000	111 050 000
MLLT4	6 962 300	7 417 700	10 587 000	22 131 000
CACYBP	13 706 000	14 912 000	18 179 000	37 841 000
PPM1G	11 319 000	9 311 900	54 347 000	107 690 000
MAP4	48 149 000	41 769 000	94 990 000	187 670 000
UBE2O	7 190 200	7 073 900	8 507 600	16 770 000
EZR	0	0	2 695 900	5 284 900
HSPE1	4 996 700	4 839 200	8 577 600	16 412 000
PC	13 363 000	14 754 000	96 240 000	179 870 000
BAZ2A	0	0	7 987 100	14 652 000
CORO7	11 112 000	10 505 000	27 276 000	49 728 000
AGFG1	83 902 000	81 336 000	122 790 000	223 830 000
EIF5	0	0	14 130 000	25 675 000
NPM1	272 950 000	257 160 000	273 050 000	496 090 000
SQSTM1	7 085 900	6 580 200	40 841 000	73 823 000
GPATCH1	19 097 000	18 403 000	19 237 000	34 736 000
OSGEP	8 767 100	13 458 000	86 897 000	156 370 000
NASP	0	0	8 531 400	15 322 000
MAP7D3	7 000 200	7 760 500	9 275 000	16 518 000
DBNL	0	0	7 379 900	13 140 000
SCRIB	0	7 433 900	12 847 000	22 558 000
COMMD10	0	0	54 611 000	95 565 000
CLINT1	8 418 000	9 065 300	16 559 000	28 648 000
UBAP2	6 231 100	5 314 100	15 672 000	26 873 000
GSPT1	29 036 000	14 323 000	47 426 000	81 049 000
CC2D1A	0	4 547 000	24 512 000	41 179 000
DOCK7	10 846 000	11 389 000	27 307 000	45 758 000
AMOT	23 310 000	23 896 000	41 313 000	68 920 000
PRRC2A	15 294 000	17 098 000	20 570 000	34 264 000
MBIP	0	0	13 569 000	22 409 000
CCDC28B	0	0	31 192 000	50 945 000
G3BP1	24 997 000	20 529 000	34 763 000	55 652 000
FLNA	52 352 000	47 146 000	88 585 000	141 720 000
LIG3	25 439 000	34 893 000	28 540 000	45 613 000
GIGYF2	23 253 000	15 272 000	125 640 000	195 310 000
EDC3	33 047 000	35 975 000	140 320 000	214 720 000
TNRC6B	14 840 000	21 165 000	30 982 000	46 936 000
CDK4	14 531 000	0	15 636 000	23 133 000
TXN	137 610 000	179 650 000	743 940 000	1 080 500 000
AP2M1	36 281 000	37 360 000	39 059 000	56 667 000
DTL	10 237 000	13 213 000	11 825 000	17 089 000
WDR70	7 999 400	8 756 000	8 098 100	11 641 000
TLN1	30 540 000	24 478 000	37 621 000	54 060 000
ATXN2L	12 590 000	13 705 000	20 873 000	29 960 000
SERPINH1	53 156 000	66 885 000	55 134 000	78 595 000
NUFIP2	10 977 000	11 117 000	26 587 000	37 625 000
SLK	7 445 800	0	7 594 100	10 741 000
ATXN2	9 181 300	7 298 900	9 289 900	13 133 000
CYLD	9 427 600	10 637 000	43 530 000	61 307 000
POP1	17 545 000	27 181 000	24 043 000	33 260 000
RANBP2	35 597 000	39 718 000	54 741 000	75 721 000
CSDE1	21 857 000	26 028 000	31 680 000	43 765 000
KDM5C	8 670 900	11 680 000	9 802 700	13 459 000
ILKAP	33 447 000	37 732 000	33 811 000	46 250 000
SPTAN1	12 326 000	10 039 000	21 082 000	28 770 000
MYL6	29 802 000	32 453 000	54 589 000	74 328 000
CDV3	0	0	33 187 000	45 051 000
FKBP4	12 139 000	14 210 000	12 365 000	16 765 000
PDAP1	4 466 800	4 408 500	24 256 000	32 848 000
SYTL4	6 055 400	7 096 500	44 382 000	60 069 000
HDLBP	131 820 000	142 080 000	133 470 000	180 040 000
WDR36	49 583 000	51 079 000	51 449 000	69 329 000
CDC37	76 355 000	84 088 000	78 462 000	105 510 000
NFX1	7 501 300	8 395 700	8 352 600	11 204 000
C14orf142	0	0	284 840 000	376 950 000
STAU1	19 860 000	19 051 000	35 545 000	46 990 000
MRPS11	14 787 000	19 672 000	16 332 000	21 567 000
RBM27	14 592 000	26 620 000	23 229 000	30 669 000
SERBP1	174 670 000	146 310 000	417 240 000	550 330 000
KIF5B	5 772 300	5 590 300	7 410 400	9 753 800
CAPZB	0	0	4 310 300	5 653 700
ACTA1	343 490 000	0	520 500 000	676 340 000
SEC23A	14 909 000	0	22 487 000	29 138 000
MRPS26	20 724 000	18 039 000	40 486 000	52 221 000
CTTN	30 748 000	23 466 000	124 470 000	160 210 000
VIM	460 420 000	390 400 000	522 480 000	671 880 000
ZNF142	6 640 400	8 130 800	12 082 000	15 408 000
DVL2	4 731 000	0	4 765 900	6 070 000
GSN	28 382 000	31 418 000	39 012 000	49 606 000
EEF2	120 030 000	108 800 000	129 000 000	163 890 000
UPF1	11 822 000	12 954 000	46 402 000	58 247 000
CAPRIN1	0	0	8 902 300	11 141 000
UBAP2L	27 531 000	22 021 000	88 092 000	110 170 000
PRIM1	44 261 000	57 409 000	56 435 000	70 570 000
RNF40	8 115 400	0	11 385 000	14 213 000
SPATA2	17 637 000	12 870 000	61 565 000	76 492 000
DHX40	16 974 000	0	18 463 000	22 840 000
EIF4G2	41 218 000	42 686 000	66 070 000	81 365 000
CCP110	0	4 111 900	4 763 000	5 859 400
TP53RK	24 439 000	19 386 000	104 420 000	128 050 000
ABCF1	25 132 000	21 427 000	30 211 000	36 904 000
DYNC1LI1	0	0	14 768 000	18 029 000
EPB41L3	8 578 100	7 444 300	9 776 400	11 914 000
ADAR	67 803 000	68 416 000	69 212 000	84 156 000
STXBP1	16 438 000	16 700 000	16 899 000	20 517 000
DDX46	90 846 000	82 672 000	94 784 000	114 940 000
NEK1	25 136 000	29 515 000	42 678 000	51 534 000
OCRL	14 165 000	14 795 000	19 516 000	23 549 000
POLD3	20 174 000	21 381 000	22 231 000	26 711 000
GPRASP2	0	0	10 690 000	12 791 000
EWSR1	60 014 000	48 890 000	64 225 000	76 828 000
RTCB	74 183 000	88 295 000	86 086 000	102 920 000
NUP93	0	0	14 980 000	17 857 000
SF3B1	110 580 000	121 730 000	117 520 000	138 690 000
RBM39	159 380 000	180 290 000	169 330 000	198 920 000
NUP214	10 886 000	11 841 000	16 749 000	19 673 000
RANBP1	45 928 000	0	77 876 000	91 404 000
NAA10	43 637 000	44 778 000	44 400 000	52 075 000
CHMP5	7 530 800	7 632 300	10 558 000	12 325 000
ESYT1	22 581 000	22 156 000	25 169 000	29 340 000
ARPC1B	0	0	15 151 000	17 628 000
PICALM	99 128 000	106 930 000	99 840 000	115 280 000
SRPRB	22 290 000	24 631 000	23 736 000	27 356 000
AHCYL2	8 947 600	9 735 400	14 471 000	16 644 000
ACTG1	3 616 100 000	5 305 300 000	4 972 400 000	5 706 400 000
HSPA5	103 850 000	100 890 000	107 500 000	123 320 000
EIF4G1	58 789 000	61 009 000	78 610 000	89 788 000
MAP2K2	28 044 000	26 630 000	28 898 000	32 922 000
FUS	196 370 000	223 680 000	221 800 000	251 530 000
SESN2	27 240 000	36 759 000	34 370 000	38 928 000
MSH2	39 412 000	45 926 000	44 481 000	50 223 000
ACTR2	18 825 000	22 139 000	24 035 000	27 083 000
MRPS31	0	22 791 000	25 566 000	28 768 000
DCD	0	4 755 400	11 693 000	13 088 000
HEATR3	6 190 100	6 853 200	8 321 800	9 307 000
DRG2	9 602 100	8 217 900	10 456 000	11 691 000
SRSF8	113 420 000	123 700 000	124 410 000	139 100 000
SUCLG1	28 453 000	27 376 000	33 994 000	37 957 000
TUBB6	0	25 315 000	26 790 000	29 800 000
RAB3GAP2	16 777 000	20 272 000	19 055 000	21 099 000
EPB41L2	28 258 000	27 962 000	28 501 000	31 554 000
GTSE1	19 033 000	14 372 000	21 089 000	23 275 000
PGAM5	39 487 000	43 883 000	40 318 000	44 441 000
EMD	40 897 000	41 977 000	43 215 000	47 551 000
CHORDC1	131 980 000	94 880 000	148 390 000	162 860 000
KIAA1279	0	0	18 502 000	20 266 000
CNOT1	45 706 000	55 241 000	53 188 000	58 152 000
HSPA8	632 860 000	634 200 000	755 310 000	825 770 000
PKP2	9 963 900	9 991 300	13 191 000	14 364 000
IGF2BP2	11 754 000	12 343 000	13 397 000	14 585 000
MAEA	25 078 000	22 591 000	25 855 000	28 144 000
EIF4B	64 393 000	47 054 000	265 340 000	288 540 000
PATZ1	23 932 000	24 446 000	25 289 000	27 484 000
PKN2	38 095 000	34 942 000	39 820 000	43 238 000
DNAJC7	40 747 000	39 366 000	41 404 000	44 934 000
MYO6	16 002 000	15 503 000	39 540 000	42 872 000
ANXA1	38 707 000	51 656 000	50 061 000	54 209 000
YTHDF1	0	10 875 000	13 081 000	14 155 000
ZC3HAV1	28 726 000	25 368 000	41 171 000	44 509 000
SFXN1	103 720 000	116 910 000	109 280 000	118 050 000
DDX50	22 928 000	24 565 000	26 670 000	28 763 000
GMPS	116 510 000	85 923 000	129 150 000	139 080 000
ZNF638	128 570 000	135 990 000	142 890 000	153 810 000
NDUFA10	37 572 000	56 024 000	56 618 000	60 857 000
PDS5B	16 176 000	19 727 000	18 651 000	20 040 000
KIAA1524	70 821 000	75 512 000	76 543 000	82 039 000
MAP7D1	29 042 000	34 862 000	36 959 000	39 606 000
TOP1	135 030 000	149 050 000	143 250 000	153 380 000
FAR1	8 107 300	6 576 200	8 439 900	9 020 400
PABPC4	26 219 000	29 570 000	29 744 000	31 754 000
RBM34	25 929 000	26 740 000	29 445 000	31 361 000
PDCD11	23 480 000	25 925 000	27 743 000	29 487 000
NSDHL	10 956 000	0	12 288 000	13 043 000
HSPH1	96 371 000	82 360 000	102 650 000	108 500 000
PCBP1	272 970 000	378 890 000	359 990 000	379 510 000
DDX24	15 935 000	19 148 000	18 401 000	19 394 000
TRIP12	21 808 000	20 824 000	22 329 000	23 525 000
MRPS2	20 763 000	22 910 000	24 707 000	26 022 000
GLS	26 590 000	23 168 000	29 471 000	31 008 000
CHD4	64 651 000	65 082 000	66 421 000	69 759 000
GTF2I	55 654 000	57 790 000	59 275 000	62 248 000
ABCE1	20 690 000	18 904 000	21 198 000	22 201 000
CUL3	6 380 200	6 076 100	6 837 400	7 158 000
LBR	147 790 000	146 230 000	162 760 000	170 160 000
RPL39	98 737 000	0	109 350 000	114 310 000
TBC1D31	13 921 000	14 257 000	14 195 000	14 836 000
DHX57	12 718 000	12 440 000	15 352 000	16 016 000
SCYL1	15 345 000	17 461 000	20 300 000	21 174 000
NAA35	74 800 000	86 932 000	97 879 000	101 930 000
RBM15	44 204 000	39 980 000	48 681 000	50 658 000
SLC25A3	268 640 000	205 930 000	285 170 000	296 690 000
ILF3	80 516 000	95 119 000	94 580 000	98 289 000
TNIK	29 131 000	24 854 000	33 747 000	34 977 000
SEC16A	39 504 000	31 284 000	47 160 000	48 847 000
NDUFB4	57 121 000	55 282 000	58 046 000	60 082 000
ZNF814	4 858 200	0	6 396 400	6 618 300
HP1BP3	12 046 000	18 119 000	18 198 000	18 812 000
PAK4	23 655 000	20 627 000	26 420 000	27 272 000
LSG1	49 900 000	49 444 000	57 824 000	59 660 000
TNRC6A	12 902 000	12 521 000	26 165 000	26 995 000
RSRC1	19 221 000	19 692 000	20 778 000	21 408 000
ARFGAP2	8 690 000	9 019 000	16 641 000	17 142 000
CAPN2	21 893 000	21 490 000	23 190 000	23 844 000
PPAN	72 191 000	70 196 000	90 876 000	93 335 000
POLD2	54 948 000	63 602 000	61 997 000	63 650 000
RBM14	1 627 400 000	1 597 100 000	1 973 700 000	2 023 300 000
PPL	0	0	118 320 000	120 950 000
RBM12B	0	0	37 006 000	37 774 000
ELAVL1	292 040 000	310 900 000	344 440 000	350 740 000
ZNF598	12 145 000	11 718 000	25 662 000	26 112 000
ANKLE2	36 296 000	36 579 000	39 573 000	40 175 000
MAGED2	64 939 000	69 064 000	76 772 000	77 814 000
TRIP13	18 234 000	18 081 000	23 027 000	23 317 000
GCN1L1	64 857 000	66 902 000	75 835 000	76 659 000
NOP58	28 594 000	32 657 000	34 191 000	34 554 000
KIF22	15 784 000	16 118 000	19 478 000	19 604 000
PABPC1	54 560 000	46 145 000	55 623 000	55 876 000
SRSF6	204 610 000	173 900 000	224 240 000	224 850 000
PHLDB1	8 574 700	8 560 000	9 945 100	9 966 800
NNT	27 704 000	19 371 000	29 875 000	29 910 000
NKRF	101 180 000	103 560 000	117 970 000	117 920 000
DIS3	47 355 000	48 960 000	51 237 000	51 137 000
HUWE1	9 505 900	9 525 600	11 544 000	11 514 000
MATR3	534 330 000	541 210 000	582 970 000	581 120 000
TMEM209	15 127 000	16 177 000	19 505 000	19 442 000
SRP72	69 673 000	66 312 000	72 885 000	72 645 000
YTHDF2	73 490 000	67 514 000	85 096 000	84 755 000
CEP350	15 092 000	23 037 000	79 505 000	79 152 000
TARS2	15 507 000	14 207 000	16 773 000	16 640 000
HNRNPM	486 840 000	453 120 000	520 100 000	515 880 000
DSP	160 230 000	165 190 000	188 460 000	186 840 000
MCAT	6 892 300	0	9 030 000	8 941 400
XAB2	35 522 000	33 440 000	48 499 000	47 970 000
TCF25	23 351 000	23 824 000	25 256 000	24 932 000
YTHDF3	33 911 000	24 832 000	42 159 000	41 580 000
XRN1	0	0	20 810 000	20 503 000
BCR	0	8 405 800	9 184 000	9 043 100
ATP6V1A	0	19 111 000	20 341 000	20 001 000
DDX6	51 573 000	36 498 000	63 547 000	62 410 000
POLR2A	15 881 000	13 326 000	19 553 000	19 187 000
CUL2	20 264 000	19 613 000	20 825 000	20 408 000
MAGED1	16 772 000	18 295 000	28 930 000	28 350 000
NOC2L	66 611 000	56 984 000	68 742 000	67 345 000
RBM15B	13 732 000	12 893 000	16 672 000	16 278 000
FAM120A	17 370 000	18 315 000	19 195 000	18 700 000
LAS1L	5 953 700	0	6 947 400	6 760 500
COPA	167 130 000	145 480 000	181 160 000	175 810 000
RBM28	33 900 000	26 642 000	33 914 000	32 900 000
FXR1	68 115 000	67 346 000	80 141 000	77 706 000
SMARCA4	13 746 000	11 130 000	13 920 000	13 497 000
PSMC3	21 157 000	0	26 489 000	25 680 000
NCAPD2	23 795 000	26 486 000	31 267 000	30 299 000
NCLN	9 112 300	7 117 200	17 396 000	16 830 000
ZNF512	140 740 000	150 720 000	163 870 000	158 520 000
C3orf17	0	5 182 500	6 517 300	6 303 900
FBL	222 640 000	216 640 000	232 600 000	224 130 000
OXSR1	31 727 000	31 268 000	34 539 000	33 132 000
IRS4	33 968 000	38 449 000	50 979 000	48 894 000
LARP1	63 074 000	60 834 000	67 929 000	64 947 000
SRSF4	19 679 000	14 202 000	19 892 000	18 981 000
ATP1A1	77 178 000	76 743 000	81 426 000	77 678 000
CLASP2	29 843 000	35 392 000	39 551 000	37 729 000
ZNF622	20 688 000	21 712 000	28 561 000	27 148 000
TRA2B	643 580 000	593 410 000	653 570 000	620 560 000
GALK1	43 624 000	44 616 000	47 350 000	44 950 000
MRPS18A	30 253 000	18 671 000	30 926 000	29 334 000
POLRMT	8 891 500	7 857 200	10 052 000	9 533 000
FASN	169 240 000	174 250 000	188 490 000	178 680 000
DDX19A	66 246 000	72 824 000	78 905 000	74 652 000
ACACA	18 050 000	16 244 000	19 732 000	18 652 000
NMD3	5 664 600	0	6 693 500	6 324 300
DHX36	130 610 000	115 650 000	150 280 000	141 990 000
PDCD6IP	316 800 000	319 280 000	354 220 000	333 970 000
ZNF721	8 284 100	9 240 300	9 991 200	9 404 100
TUBA1B	5 612 900 000	5 471 300 000	5 958 500 000	5 605 700 000
CEP85	0	29 075 000	46 034 000	43 276 000
IGF2BP1	603 200 000	573 030 000	654 090 000	613 880 000
PHKA1	25 347 000	29 157 000	32 273 000	30 281 000
DDX23	19 715 000	17 667 000	21 290 000	19 968 000
RPL26	254 640 000	225 050 000	272 920 000	255 400 000
KPNA1	77 430 000	71 835 000	88 704 000	83 009 000
ATXN10	17 651 000	0	19 384 000	18 128 000
TRIP4	0	10 865 000	14 049 000	13 127 000
KIF2C	9 816 500	8 753 200	14 957 000	13 953 000
RAD50	8 124 300	7 255 200	12 076 000	11 247 000
CCDC59	91 841 000	91 338 000	99 630 000	92 746 000
GPS2	0	0	12 503 000	11 614 000
NOA1	21 571 000	21 447 000	32 818 000	30 447 000
MRPS18B	64 404 000	67 464 000	76 711 000	71 119 000
HNRNPU	4 740 100 000	4 939 600 000	5 340 800 000	4 949 100 000
MRPS5	27 658 000	28 576 000	34 255 000	31 698 000
TUBB8	438 270 000	459 490 000	506 200 000	467 920 000
VRK1	62 960 000	67 410 000	78 165 000	72 122 000
MRPL4	65 669 000	61 822 000	71 076 000	65 493 000
RPS19BP1	0	28 153 000	47 735 000	43 897 000
EIF4H	53 913 000	57 774 000	77 754 000	71 364 000
RPL24	784 120 000	823 660 000	898 770 000	824 000 000
CDK9	122 000 000	84 478 000	135 950 000	124 560 000
SUN1	0	0	8 854 400	8 098 000
PRPF31	37 436 000	33 883 000	43 380 000	39 642 000
PRKRA	14 305 000	13 831 000	15 541 000	14 183 000
MRPS34	58 814 000	55 142 000	76 041 000	69 320 000
TUBGCP2	20 889 000	14 613 000	28 484 000	25 959 000
ALG1	0	0	151 750 000	138 050 000
AP2A2	10 036 000	10 936 000	12 899 000	11 705 000
AIMP2	59 416 000	0	64 911 000	58 750 000
RBMX	406 520 000	393 170 000	465 300 000	421 110 000
MRPS22	230 700 000	221 360 000	249 880 000	225 680 000
SMARCB1	43 409 000	46 225 000	52 219 000	47 063 000
RDH11	57 425 000	51 725 000	62 436 000	56 154 000
ABL2	8 903 700	0	9 014 600	8 105 500
NSA2	169 180 000	186 750 000	209 360 000	187 590 000
YTHDC2	23 648 000	24 619 000	30 191 000	27 007 000
PGD	13 288 000	12 183 000	14 415 000	12 885 000
HLTF	20 868 000	22 358 000	26 566 000	23 712 000
RNPS1	70 856 000	37 669 000	76 177 000	67 711 000
REEP4	10 370 000	8 562 400	11 236 000	9 925 300
MARS	122 080 000	130 420 000	148 040 000	130 760 000
NVL	30 398 000	27 230 000	32 614 000	28 803 000
RPS3A	248 630 000	198 430 000	273 810 000	241 780 000
EIF3A	323 010 000	277 170 000	348 090 000	307 270 000
VDAC3	50 816 000	54 067 000	69 587 000	61 369 000
EIF2B3	30 415 000	27 842 000	31 712 000	27 950 000
KIF1C	6 541 400	0	8 426 800	7 411 300
TCHP	49 605 000	55 370 000	68 108 000	59 794 000
HSPA9	32 762 000	22 752 000	35 913 000	31 520 000
SULT1A1	19 364 000	21 273 000	26 277 000	23 044 000
ERAL1	15 236 000	0	16 563 000	14 493 000
POLR2B	19 187 000	18 284 000	22 111 000	19 319 000
USP9X	5 045 200	5 440 900	6 895 000	6 021 900
CSK	28 188 000	27 775 000	34 838 000	30 401 000
NOM1	21 801 000	20 476 000	26 003 000	22 569 000
RBM4B	56 591 000	48 136 000	63 578 000	55 045 000
TTF2	0	0	3 960 700	3 419 000
PUM1	158 980 000	177 370 000	212 080 000	183 010 000
HAUS5	42 820 000	42 453 000	52 209 000	45 002 000
NBN	9 711 700	0	12 484 000	10 737 000
TBL2	23 853 000	0	26 327 000	22 588 000
GPR50	21 104 000	21 285 000	24 972 000	21 415 000
PFKM	21 068 000	24 504 000	37 659 000	32 159 000
VPS33B	33 699 000	0	46 091 000	39 340 000
TFIP11	32 568 000	28 074 000	34 227 000	29 164 000
KPNA2	151 660 000	129 070 000	192 080 000	163 490 000
MCMBP	78 826 000	71 085 000	86 641 000	73 515 000
NUP155	145 670 000	155 380 000	197 630 000	167 640 000
DDX47	23 400 000	26 505 000	31 793 000	26 966 000
CRNKL1	11 704 000	11 089 000	13 555 000	11 492 000
DUSP11	58 770 000	51 234 000	80 269 000	68 046 000
NOC3L	50 290 000	46 970 000	57 058 000	48 296 000
CDC27	24 850 000	21 015 000	30 025 000	25 365 000
CRTC3	18 771 000	18 676 000	25 203 000	21 277 000
CXXC1	22 210 000	17 941 000	23 582 000	19 907 000
C19orf52	7 768 500	6 331 000	21 519 000	18 143 000
KPNB1	13 730 000	12 093 000	14 633 000	12 330 000
YWHAH	20 427 000	0	23 690 000	19 898 000
CCDC127	28 462 000	22 509 000	29 556 000	24 810 000
CBS	14 656 000	12 588 000	22 962 000	19 256 000
RPS14	137 280 000	125 030 000	159 240 000	133 350 000
UTP18	43 194 000	36 556 000	45 565 000	38 091 000
BRIX1	43 217 000	40 322 000	50 345 000	42 085 000
DDX54	53 150 000	50 904 000	75 183 000	62 632 000
CLK1	29 515 000	28 095 000	43 773 000	36 296 000
SMARCD2	12 904 000	0	17 731 000	14 681 000
ATAD3A	55 918 000	55 059 000	73 610 000	60 411 000
SRSF7	159 030 000	154 750 000	192 880 000	158 240 000
TAOK2	28 473 000	28 243 000	36 833 000	30 145 000
MED24	0	5 103 600	6 710 100	5 479 600
LRBA	12 486 000	9 239 100	13 400 000	10 925 000
FAM98A	102 420 000	68 128 000	109 270 000	88 964 000
EXOC8	0	0	13 447 000	10 885 000
ORC4	30 756 000	27 704 000	39 396 000	31 792 000
AMPD2	22 951 000	10 453 000	24 892 000	20 030 000
TONSL	19 749 000	18 886 000	26 860 000	21 586 000
CCT6A	50 759 000	38 132 000	61 040 000	48 885 000
LIG1	0	0	7 270 700	5 812 400
ABLIM1	22 681 000	20 431 000	26 350 000	21 015 000
FLII	20 310 000	24 335 000	33 428 000	26 600 000
NAT10	106 060 000	89 587 000	126 390 000	100 400 000
ITCH	23 525 000	22 594 000	31 696 000	25 163 000
RPL30	15 339 000	13 263 000	18 869 000	14 956 000
SNX4	33 467 000	29 723 000	40 741 000	32 266 000
CLASRP	330 840 000	267 390 000	370 040 000	292 620 000
UGDH	22 470 000	18 860 000	24 470 000	19 332 000
ARHGEF2	12 700 000	0	19 024 000	14 972 000
SPTBN2	21 399 000	16 986 000	26 823 000	21 099 000
EXD2	10 685 000	11 467 000	15 953 000	12 547 000
TJP2	25 233 000	26 591 000	35 631 000	28 010 000
CHD2	0	0	27 759 000	21 794 000
EXOC5	30 814 000	25 482 000	36 394 000	28 295 000
MRPL47	37 600 000	31 332 000	46 278 000	35 949 000
MRPS14	435 060 000	377 810 000	505 800 000	390 540 000
CHD1L	17 905 000	20 435 000	27 838 000	21 451 000
LEMD3	0	0	9 673 100	7 442 000
THOC2	12 678 000	10 822 000	19 028 000	14 638 000
CYFIP1	33 684 000	29 326 000	43 180 000	33 038 000
TWF1	7 666 900	6 907 500	9 466 000	7 228 500
TIPRL	11 496 000	0	16 185 000	12 300 000
DARS	35 430 000	28 760 000	40 523 000	30 559 000
HOXA5	53 536 000	52 936 000	76 806 000	57 748 000
ZBTB11	15 740 000	0	22 890 000	17 161 000
AGPS	31 583 000	24 696 000	39 620 000	29 359 000
ARGLU1	43 954 000	23 956 000	53 149 000	39 034 000
NEDD4	6 318 400	0	8 102 000	5 943 800
RAD21	0	0	16 247 000	11 817 000
ECI2	32 959 000	22 688 000	35 668 000	25 576 000
DIAPH3	12 368 000	10 070 000	20 447 000	14 526 000
NTMT1	34 305 000	33 635 000	48 399 000	34 026 000
ZAK	16 005 000	11 435 000	20 599 000	14 377 000
PEX16	11 945 000	0	18 063 000	12 592 000
PLAT	4 137 800	4 117 400	5 958 900	4 148 000
YARS2	8 680 800	0	13 429 000	9 155 400
SMG7	11 054 000	10 143 000	16 528 000	11 196 000
HIST1H3A	936 520 000	575 330 000	1 017 800 000	670 310 000
NPEPPS	9 139 200	4 454 000	11 071 000	7 254 500
GNL1	38 773 000	0	48 950 000	31 450 000
MYH10	472 190 000	262 140 000	731 310 000	461 700 000
METTL13	0	0	13 451 000	8 450 700
MYH9	45 184 000	29 093 000	92 177 000	56 224 000
CDC20	0	0	20 058 000	10 029 000
RCC2	19 322 000	19 899 000	49 033 000	19 983 000
HECTD1	8 161 200	4 968 400	14 404 000	5 208 900

To validate the association between LAGE3 and MRE11, we performed both proximity ligation assay (PLA) and Co-IP with either ectopic expression of FLAG-LAGE3 or endogenous LAGE3, which confirmed the spatial proximity and physical interaction between the two proteins (Fig. 4C and D and Supplementary Fig. S4C–E). Human LAGE3 and its orthologs in other species contain a conserved PCC1 domain at its C-termius, along with an additional N-terminal extension (N1 region) unique to higher eukaryotes. Co-IP assay demonstrates that LAGE3 interacts with MRE11 specifically through the conserved PCC1 domain (Supplementary Fig. S4F and G). And its N-terminal domain supports the stability of intact LAGE3 protein.

Previous works of ours and others have demonstrated that MRE11 mediates the nascent strand DNA degradation at stalled replication forks [23]. Therefore, this raised the possibility that LAGE3 safeguards stalled forks by antagonizing MRE11 activity. To test this, we performed SIRF analysis targeting MRE11 and observed a dramatic increase in replication fork-associated MRE11 in LAGE3-deficient cells with HU treatment (Fig. 4E). Notably, treatment with mirin, a small-molecule inhibitor of MRE11, successfully restores fork stability in LAGE3-deficient cells, as shown by DNA fiber assay (Fig. 4F).

Together, these results suggest that LAGE3 protects stalled forks from aberrant degradation by limiting MRE11 access, thereby preserving fork integrity under replication stress.

### Other KEOPS complex components contribute to stalled fork protection

**Figure 5.**
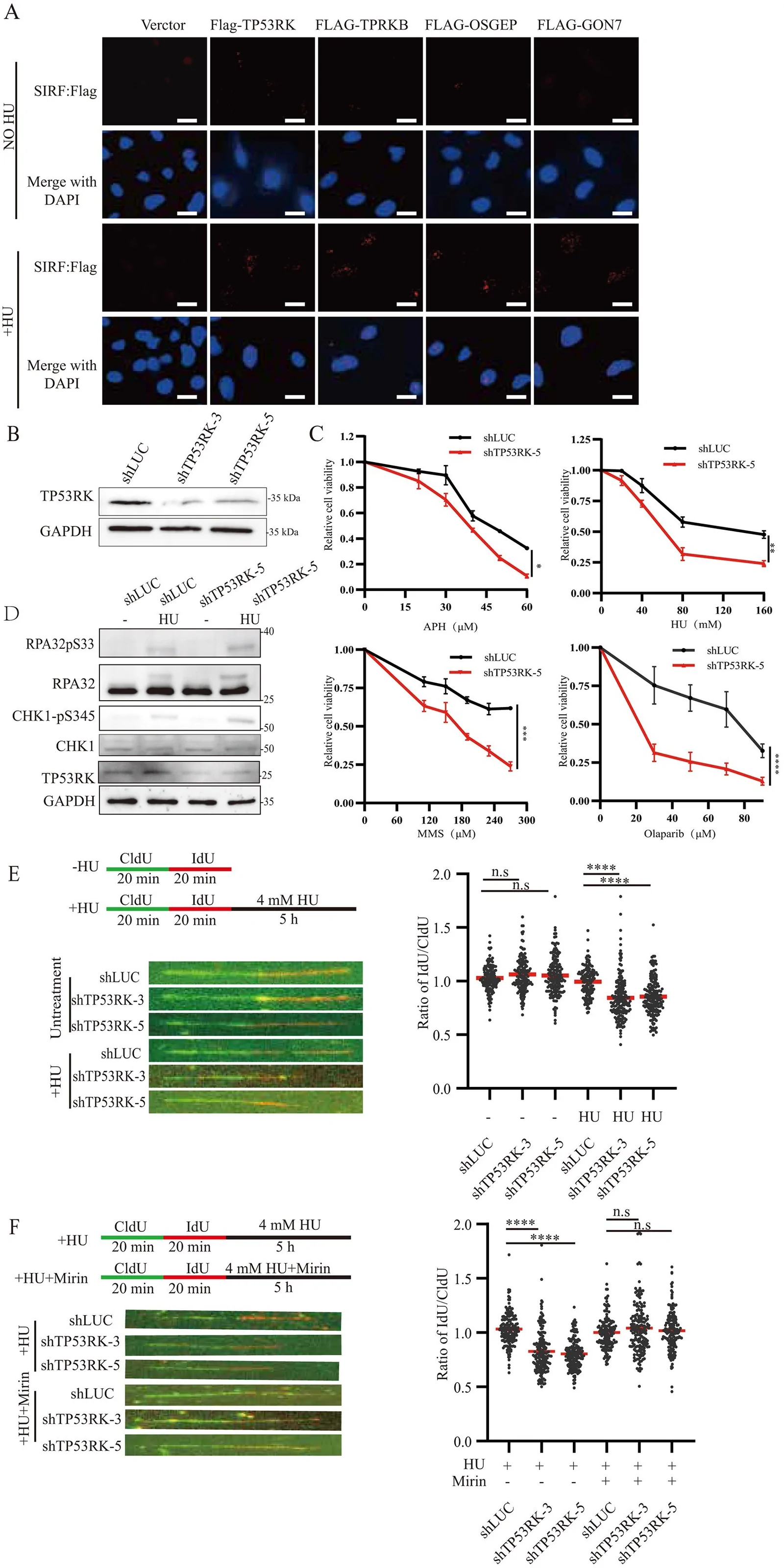
Other subunits of KEOPS complex contribute to stalled fork protection. (**A**) Representative images of SIRF assay showing the localization of TP53RK, TPRKB, OSGEP, and GON7 at active and stalled forks. Scale bars: 20 μm. The protein expression and SIRF foci quantification were included in Supplementary Fig. S5A and B. (**B**) Western blot showing TP53RK knock-down in U2OS cells. (**C**) CCK-8 assay showing the cell sensitivity to different replication stressors with indicated concentrations for TP53RK-deficient cells. The data were collected from three independent experiments. Statistical analysis was performed with two-way ANOVA, *P < .05, **P < .01, ***P < .001, ^****^P < .0001. Error bars: SEM. (**D**) TP53RK knockdown increases the RPA32pS33 and CHK1pS345 expression in response to DNA replication stress. (**E**) DNA fiber analysis of the stability of replication forks with and without HU treatment in TP53RK-deficient cells. The ratio of IdU/CldU was used to evaluate the stability of nascent stranded DNA. Data were collected from two biological repeats and statistical analysis was performed with one-way ANOVA, ^****^P < .0001, Error bars: SEM. (**F**) DNA fiber analysis of the stability of stalled replication forks with and without MRE11 inhibitor mirin treatment in TP53RK-deficient cells. The ratio of IdU/CldU was used to evaluate the stability of nascent stranded DNA. Data were collected from two biological repeats and statistical analysis was performed with one-way ANOVA, ^****^P < .0001, Error bars: SEM.

Whole-exome sequencing (WES) in GAMOS patients has revealed pathogeneic mutations in all five genes encoding KEOPS complex subunits, implying that the biological functions of KEOPS require its structural and functional integrity. To examine whether the entire KEOPS complex is essential for protecting stalled forks, we analyzed the localization of other KEOPS subunits in response to DNA replication stress. U2OS cells were engineered to stably express FLAG-tagged TP53RK, TPRKB, OSGEP, and GON7, respectively. SIRF assays showed that all four KEOPS subunits, like LAGE3, localized to stalled replication forks under HU treatment (Fig. 5A and Supplementary Fig. S5A and B).

Structural studies of the KEOPS complex reveal that LAGE3 occupies a center position in the crystallized OSGEP-LAGE3-GON7 subcomplex, mediating the connection between OSGEP and GON7 [9]. Meanwhile, TP53RK and TPRKB form a heterodimer (PDB: 6WQX) and hydrolyze ATP to AMP, and interact with the 3′ CCA tail of tRNA. Given this context, we further investigated the role of TP53RK in regulating stalled forks. The endogenous TP53RK was knocked down by lentivirus-mediated transduction using two different shRNAs, and knockdown efficiency was verified by western blotting (Fig. 5B). Similar to LAGE3 knockdown, the lack of TP53RK reduced cell proliferation, rendered cells more sensitive to DNA replication stressors, and elevated genomic instability, characterized by micronuclei formation (Fig. 5C and Supplementary Fig. S5C and D). Consistently, immunoblotting analysis showed heightened phosphorylation of ATR pathway effectors RPA32pS33 and CHK1pS345 under HU treatment in TP53RK-deficient cells (Fig. 5D). Immunofluorescence analysis further demonstrated the increased γH2AX levels and enhanced chromatin-bound RPA32 following DNA replication stress in the condition of TP53RK deficiency (Supplementary Fig. S5E–H).

Finally, the DNA fiber assay indicated that TP53RK deficiency led to nascent strand DNA degradation under replication stress, similar to the effects observed with LAGE3 depletion. The degradation was reversed upon treatment with mirin, suggesting that, like LAGE3, TP53RK also protects stalled forks by limiting MRE11 activity (Fig. 5E and F).

Collectively, these results demonstrate that multiple components of the KEOPS complex, including LAGE3 and TP53RK, localize to stalled replication forks and function cooperatively to maintain fork stability under replication stress. This underscores the requirement for an intact KEOPS complex in safeguarding genome integrity.

### Purified KEOPS complex directly binds to ds-ssDNA junction and inhibits MRE11 nuclease activity

The 3′–5′ exonuclease activity of MRE11 enables degradation of the regressed arm of reversed replication forks, thereby promoting fork destabilization under replication stress [33]. Given that yeast ScKEOPS exhibits binding activity to both ssDNA and dsDNA substrates, we hypothesized that the human KEOPS complex may bind ds-ssDNA junctions. These structures mimic stalled replication forks and thereby inhibit MRE11-mediated resection.

**Figure 6.**
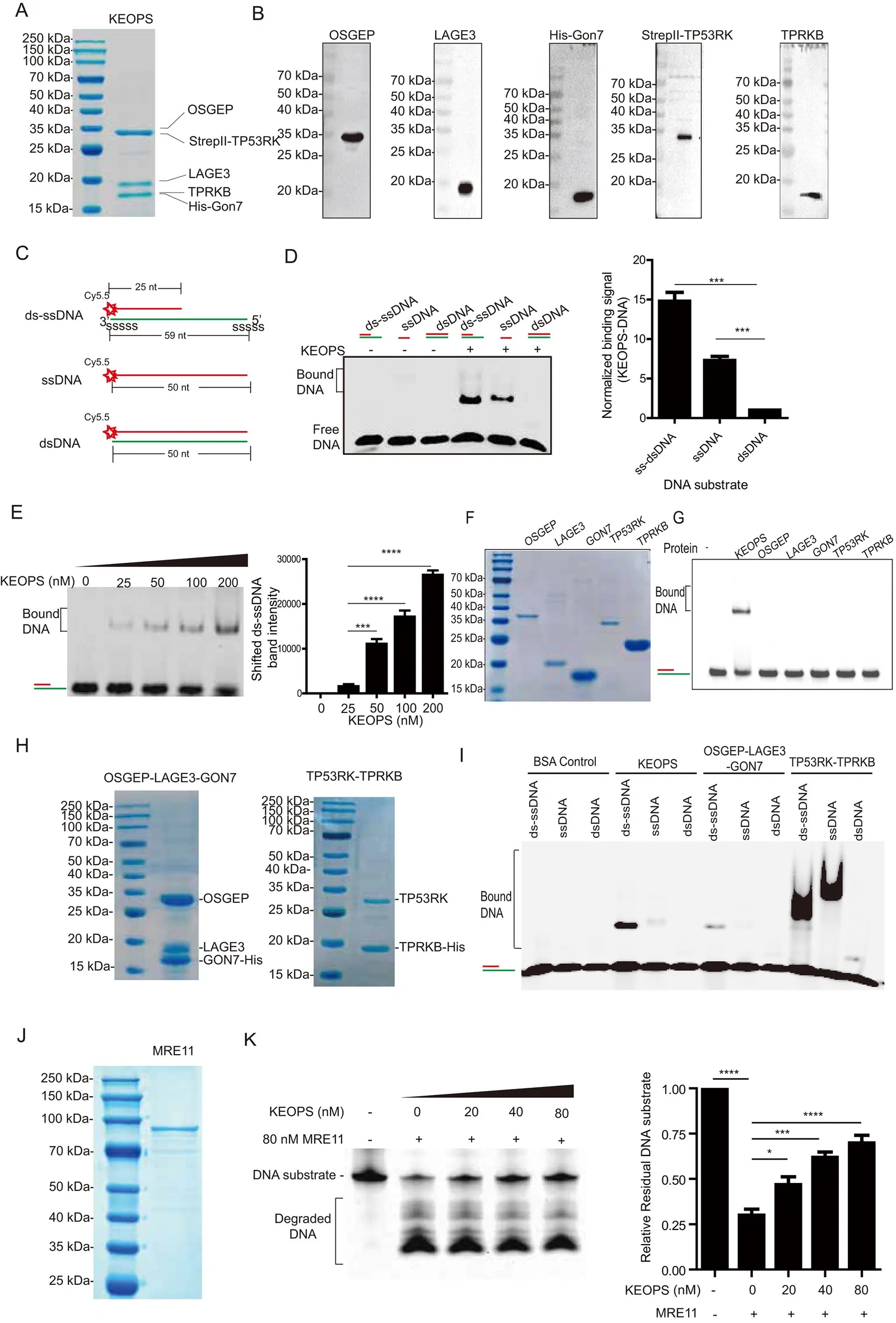
KEOPS complex directly binds DNA substrate to prevent its degradation by MRE11. (**A**) Coomassie blue-stained sodium dodecyl–sulfate polyacrylamide gel electrophoresis (SDS–PAGE) gel of purified human KEOPS complex. (**B**) Western blot showing the expressed subunits of purified human KEOPS complex. (**C**) Schematic of 5’ Cy5.5-labeled ds-ssDNA, dsDNA, and ssDNA substrates. The nucleotide with phosphorothioate modification on both ends of ds-ssDNA was referred to as “S”. (**D**) Binding analysis of different types of DNA (ds-ssDNA, ssDNA, and dsDNA) to KEOPS complex. The 5′ Cy5.5-labeled ds-ssDNA, ssDNA, and dsDNA substrates were incubated with equal amount of KEOPS complex, and samples were analyzed by 4% polyacrylamide gel. The results are graphed, and data were collected from three biological repeats and statistical analysis was performed with one-way ANOVA, ***P < .001, Error bars: SEM. (**E**) Binding analysis of ds-ssDNA to the indicated concentration of the KEOPS complex. Cy5.5-labeled ds-ssDNA substrates were incubated with the indicated concentration of purified KEOPS complex. Samples were analyzed by 4% polyacrylamide gel. The results are graphed, and data were collected from three biological repeats and statistical analysis was performed with one-way ANOVA, ***P < .001, ^****^P < .0001, Error bars: SEM. (**F**) Coomassie blue-stained SDS–PAGE gel (12%) of purified human KEOPS components individually. (**G**) Binding analysis of ds-ssDNA to the KEOPS complex and its subunits. Equal amount of each subunit or complex was incubated with ds-ssDNA substrate. (**H**) Coomassie blue-stained SDS–PAGE gel (12%) of purified subcomplex OSGEP-LAGE3-GON7 and TP53RK-TPRKB. (**I**) Binding analysis of ds-ssDNA to the KEOPS complex and its two subcomplexes, OSGEP-LAGE3-GON7 and TP53RK-TPRKB. Equal amount of protein complexes (200 nM) was incubated with ds-ssDNA, ssDNA and dsDNA substrates, and then samples were analyzed on 4% polyacrylamide gel. (**J**) Coomassie blue-stained SDS–PAGE gel of purified human MRE11. (**K**) KEOPS complex binds ds-ssDNA to limit MRE11 nuclease activity. Samples were resolved in 27% denatured polyacrylamide gel. Residual DNA substrates were quantified. The data were collected from three biological repeats and statistical analysis was performed with one-way ANOVA, ^****^P < .0001, ***P < .001, *P < .05, Error bars: SEM.

To test this, we purified the intact human KEOPS complex using an *E. coli* expression system, validating the successful expression of all the components using the specific antibodies via immunoblotting (Fig. 6A and B and Supplementary Fig. S6A). Subsequently, we assessed the binding affinity for KEOPS to Cy5.5-labeled ds-ssDNA junction, ssDNA, and dsDNA substrates using electrophoretic mobility shift assays (EMSA) (Fig. 6C and D). Unlike yeast ScKEOPS, which preferentially binds to all the ds-ssDNA, ssDNA, and dsDNA substrates, the human KEOPS complex possesses far stronger binding affinity for ds-ssDNA junctions compared to ssDNA, whereas it barely interacts with dsDNA (Fig. 6D and Supplementary Fig. S6B and C). These results suggest that, under replication stress, extended ssDNA regions at stalled forks may recruit the KEOPS complex. The divergence in DNA-binding preferences between human and yeast KEOPS implies an evolutionary adaptation in higher eukaryotes, favoring ssDNA overhang structures such as double-strand breaks (DSBs), stalled replication forks, and telomeres.

The ds-ssDNA substrates effectively mimic the branched DNA intermediates formed during replication fork reversal. We found that the KEOPS complex binds to it in a concentration-dependent manner (Fig. 6E). To determine whether individual subunits mediate this binding or require the full KEOPS complex, we purified each of the five KEOPS subunits individually (Fig. 6F). Interestingly, EMSA results showed that individual subunits barely bind to ds-ssDNA junctions (Fig. 6G). Previous studies indicated that OSGEP-LAGE3-GON7 and TP53RK-TPRKB could form stable subcomplexes; we therefore purified the two human KEOPS subcomplexes (Fig. 6H). Compared to the intact KEOPS complex, EMSA results showed that OSGEP-LAGE3-GON7 subcomplex displayed much weaker binding affinity for ds-ssDNA and ssDNA, while TP53RK-TPRKB assembly possessed elevated binding affinity for DNA substrates with retarded band shift. However, neither the intact human KEOPS complex nor any of its subcomplexes displays appreciable binding affinity for dsDNA (Fig. 6I).

To further elucidate the functional association between KEOPS and MRE11 during replication fork resection, we obtained MRE11 nuclease protein from HEK293T cells and co-incubated it with KEOPS using ds-ssDNA as substrate (Fig. 6J). Notably, the KEOPS complex partially inhibited MRE11-mediated cleavage of the ds-ssDNA junctions, providing direct biochemical evidence to support our cellular findings that LAGE3 stabilizes stalled forks by inhibiting MRE11 nuclease activity (Fig. 6K).

### GAMOS-associated LAGE3 mutations exacerbate genome instability under DNA replication stress

**Figure 7.**
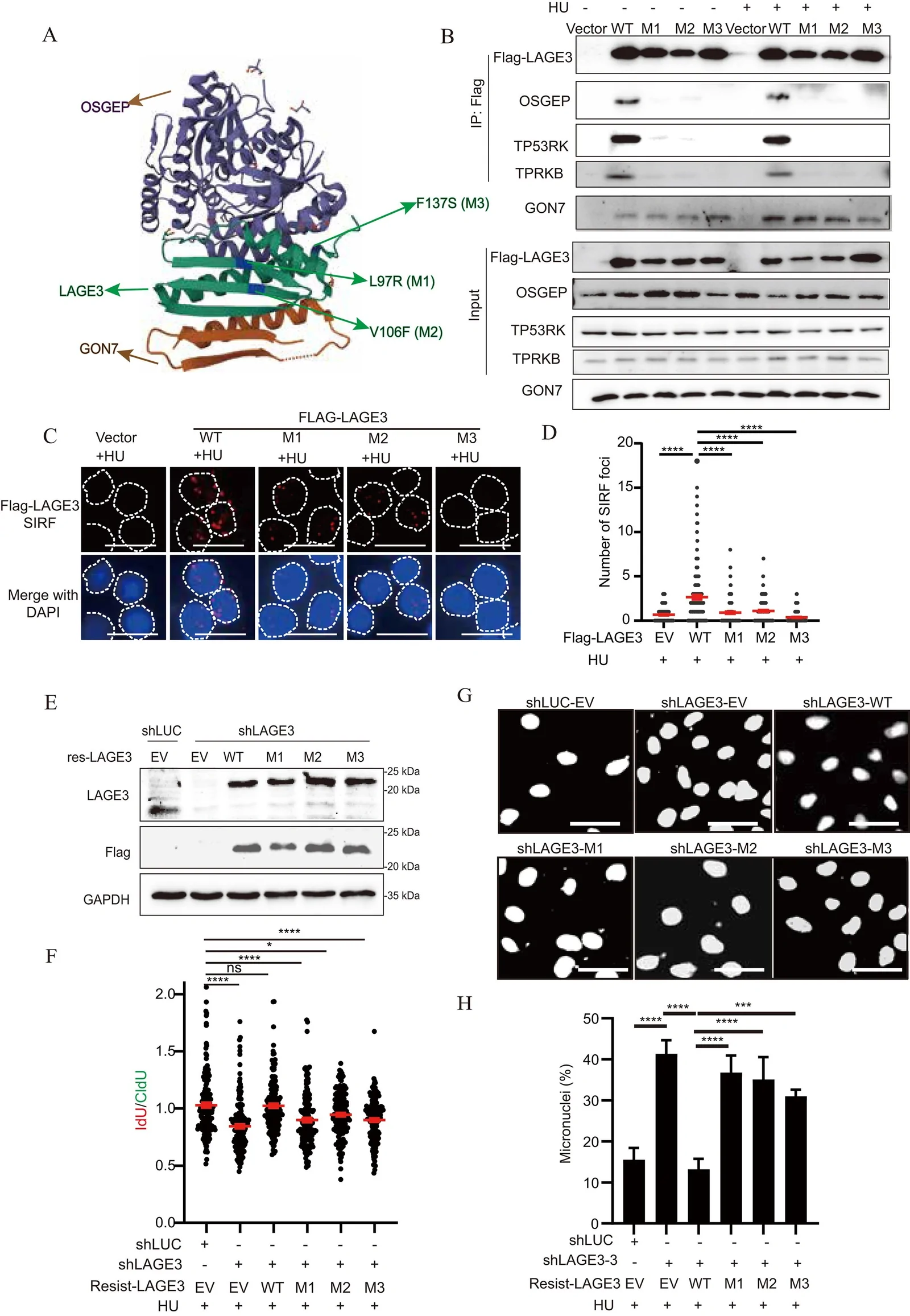
GAMOS disease-associated LAGE3 mutations exacerbate genome instability under DNA replication stress. (**A**) The crystal structure of subcomplex of OSGEP-LAGE3-GON7. The GAMOS disease-associated LAGE3 mutations (L97R, V106F, and F137S) were labeled. (**B**) CoIP showing the interaction between Flag-LAGE3 mutants and other components of KEOPS. (**C**) Detection of LAGE3 wild type (WT) and mutants on stalled and active forks with SIRF assay. Representative SIRF images are shown. Scale bar: 20 μm. (**D**) Quantifying the SIRF foci number per nucleus of FLAG-LAGE3 WT and mutations in panel (C). (**E**) Western blot showing the endogenous LAGE3 knockdown and the ectopic expression of RNAi-resistant LAGE3 WT and mutations in HeLa cells. (**F**) DNA fiber analysis of nascent-strand DNA degradation with LAGE3 mutations expression. Data were collected from two biological repeats and statistical analysis was performed with one-way ANOVA, ^****^P < .0001, ***P < .001, *P < .05. Error bars: SEM. (**G, H**). Micronuclei were stained with DAPI in HeLa cells with LAGE3 knockdown, and RNAi-resistant LAGE3 WT and mutations expression with concurrent knockdown of endogenous LAGE3. The percentage of cells with micronuclei was quantified from three independent experiments. *P* values were calculated by one-way ANOVA analysis, ***P < .001, ^****^P < .0001. Error bars: SEM.

Our results have indicated that LAGE3 deficiency leads to elevated genome instability in response to DNA replication stress. To assess whether GAMOS-associated LAGE3 mutations also impair genome maintenance, we examined three point mutations—L97R, V106F, and F137S—previously identified in GAMOS patients (designated as M1, M2, and M3, respectively; Fig. 7A) [2, 34]. Based on the available crystal structure of the GON7-LAGE3-OSGEP, M1 and M2 mutations of LAGE3 do not localize on the interfaces with GON7 and OSGEP. In contrast, the M3 mutation on the C-terminus of LAGE3 localizes on the interface of LAGE3 and OSGEP.

Co-immunoprecipitation assays showed that all three LAGE3 mutants disrupted KEOPS complex assembly (Fig. 7B). Given that LAGE3 binding to ds-ssDNA junctions depends on KEOPS formation, we next evaluated the ability of these mutants to localize to stalled forks. SIRF assays demonstrated significantly reduced fork localization for all three mutants compared to wild-type LAGE3 (Fig. 7C and D).

To assess the functional impact of these mutations on replication fork stability and genome integrity, we stably expressed RNAi-resistant forms of wild-type (WT) and mutant LAGE3 (M1–M3) in shLAGE3 knockdown HeLa cells, thereby minimizing compensation by endogenous LAGE3 (Fig. 7E). Upon treatment with hydroxyurea (HU) to induce replication stress, immunofluorescence staining revealed increased γH2AX and RPA32 signals in cells expressing the LAGE3 mutants, indicative of elevated DNA damage and ssDNA accumulation (Supplementary Fig. S7A and D). DNA fiber assays showed that none of the mutants could protect stalled forks from degradation (Fig. 7F). Consistent with this, the frequency of micronuclei was significantly elevated in both the shLAGE3 and LAGE3 mutant-expressing cells compared to controls expressing shLUC or WT LAGE3 (Fig. 7G and H).

Together, these results suggest that GAMOS-associated LAGE3 mutations impair KEOPS function at stalled forks, leading to increased genome instability, and may thereby contribute to the pathogenesis of GAMOS.

## Disscussion

GAMOS is a severe and rare disorder caused by mutations in genes encoding components of the KEOPS complex, as well as several other loci [2, 9, 35]. The canonical role of the KEOPS complex in catalyzing the t6A RNA modification is well established across mammals, plants, and archaea [2, 6, 29]. Recently, we demonstrated that the KEOPS complex also contributes to the protection of stalled replication forks under DNA replication stress. Here, we provide mechanistic insight into the function of LAGE3, a core KEOPS subunit, in safeguarding stalled fork integrity. We show that loss of LAGE3 induces spontaneous genomic instability and hypersensitivity to DNA replication stress. SIRF assays reveal LAGE3 localization at the stalled forks, while TurboID-based proximal proteomics identifies the nuclease MRE11 as a LAGE3-interacting protein. Importantly, inibition of MRE11 nuclease activity rescues fork stability in LAGE3-deficient cells. Other KEOPS subunits also localize to stalled forks, and TP53RK in particular protects fork integrity by suppressing aberrant MRE11-mediated degradation. Moreover, GAMOS-associated LAGE3 mutations further exacerbate genomic instability following DNA replication stress. Collectively, these findings highlight an essential role for LAGE3 in stalled fork protection and suggest that genome instability might contribute to GAMOS pathogenesis.

Beyond t6A modification, the KEOPS complex regulates several aspects of chromosomal DNA metabolism [3, 4, 8]. Genome-wide screening in *S. cerevisiae* identified KEOPS as a suppressor of the growth defects of *cdc13* mutants, implicating KEOPS in telomere maintenance [3]. Subsequent work showed that KEOPS promotes G-overhang formation independently of t6A modification [4]. Given the central role of telomere length in cellular senescence, these findings suggest that KEOPS may influence organismal aging. Biochemical assays further demonstrate that KEOPS binds not only tRNA but also ssDNA and dsDNA [8]. Moreover, KEOPS deficiency impairs homologous recombination at DSBs, where KEOPS subunits restrict the activities of EXO1 and DNA2 to limit excessive end resection. However, these functions have been characterized primarily in *S. cerevisiae*, and whether similar mechanisms operate in mammalian cells and how they relate to GAMOS remains largely unexplored.

Because a stalled replication fork resembles extended ssDNA or ss-dsDNA junctions found at DSBs, many DNA repair proteins, including CST, RPA, and RAD51, function in both DNA replication stress responses and classical DNA repair to maintain replication fidelity [15, 23, 36, 37]. Consistent with these factors, the KEOPS complex binds ssDNA and ds-ssDNA junctions across species. *In vitro* purified human KEOPS protects ds–ssDNA junctions from MRE11 digestion. Based on this, we propose that KEOPS complex binds ssDNA at stalled forks and induces structural changes that occlude MRE11 recognition or access. In human podocytes, knockdown of KEOPS subunits activates DDR, followed by apoptosis [2], suggesting that replication fork collapse may underlie the observed DNA damage. The nuclear colocalization of TP53RK with PARP1 in renal glomeruli, along with their interaction detected by co-immunoprecipitation, further supports a role for KEOPS in replication stress response. Because depletion of OSGEP or TPRKB also induces genomic instability, we infer that full KEOPS integrity is essential for replication fork protection.

Genomic instability is a hallmark of tumorigenesis, premature aging, and neurodegenerative disorders. KEOPS subunits have been implicated in multiple cancers, including hepatocellular carcinoma [22], lung cancer [38], colorectal cancer [19], breast cancer [39], and papillary thyroid carcinoma [20]. Individuals carrying KEOPS mutations exhibit primary microcephaly, developmental delay, and seizures—phenotypes frequently associated with defects in DNA damage response genes such as ATM, ATR, MCPH1, and CHK2. We hypothesize that KEOPS mutations promote tumorigenesis and neurological disease by disrupting telomere regulation, DSB repair, and replication stress responses, ultimately driving genomic instability.

Although accumulating evidence indicates that KEOPS binds chromosomal DNA and maintains genome stability, several gaps remain. First, although the KEOPS-tRNA binding interface has been structurally defined in archaea, it is unknown whether the KEOPS-DNA binding mode resembles its tRNA binding architecture [9, 40, 41]. Defining the KEOPS-DNA structureal interface will clarify how KEOPS mutations cause GAMOS. Second, although GAMOS is attributed primarily to defective t6A modification, it remains unclear whether impaired replication stress responses and DNA repair also contribute to disease progression. Third, in addition to GAMOS, KEOPS is linked to multiple cancers; however, whether its DNA-related functions directly participate in carcinogenesis remains unresolved. Thus, further investigation into both t6A-dependent and chromosomal DNA-protective functions of the KEOPS complex will be critical for identifying therapeutic targets for GAMOS and KEOPS-associated cancers.

## Supplementary Material

gkag842_Supplemental_File

## Data Availability

The data underlying this article are available in the article and in its Supplementary material. All the raw data have been deposited in ProteomeXchange (ID: PXD066518). Further information and requests for resources and reagents should be directed to and will be fulfilled by the Contacts, xxlv@sdfmu.edu.cn (X.L.).
