# Supplementary material for: L Antigen Family Member 3 stabilizes stalled replication forks by reducing MRE11 resection to maintain genome stability
Source: Nucleic Acids Res. 2026 Aug 24;54(16):gkag842. doi: 10.1093/nar/gkag842 (PMC13501129; doi:10.1093/nar/gkag842)
Supplement: Supplementary file 1 [file gkag842_supplemental_file.pdf]

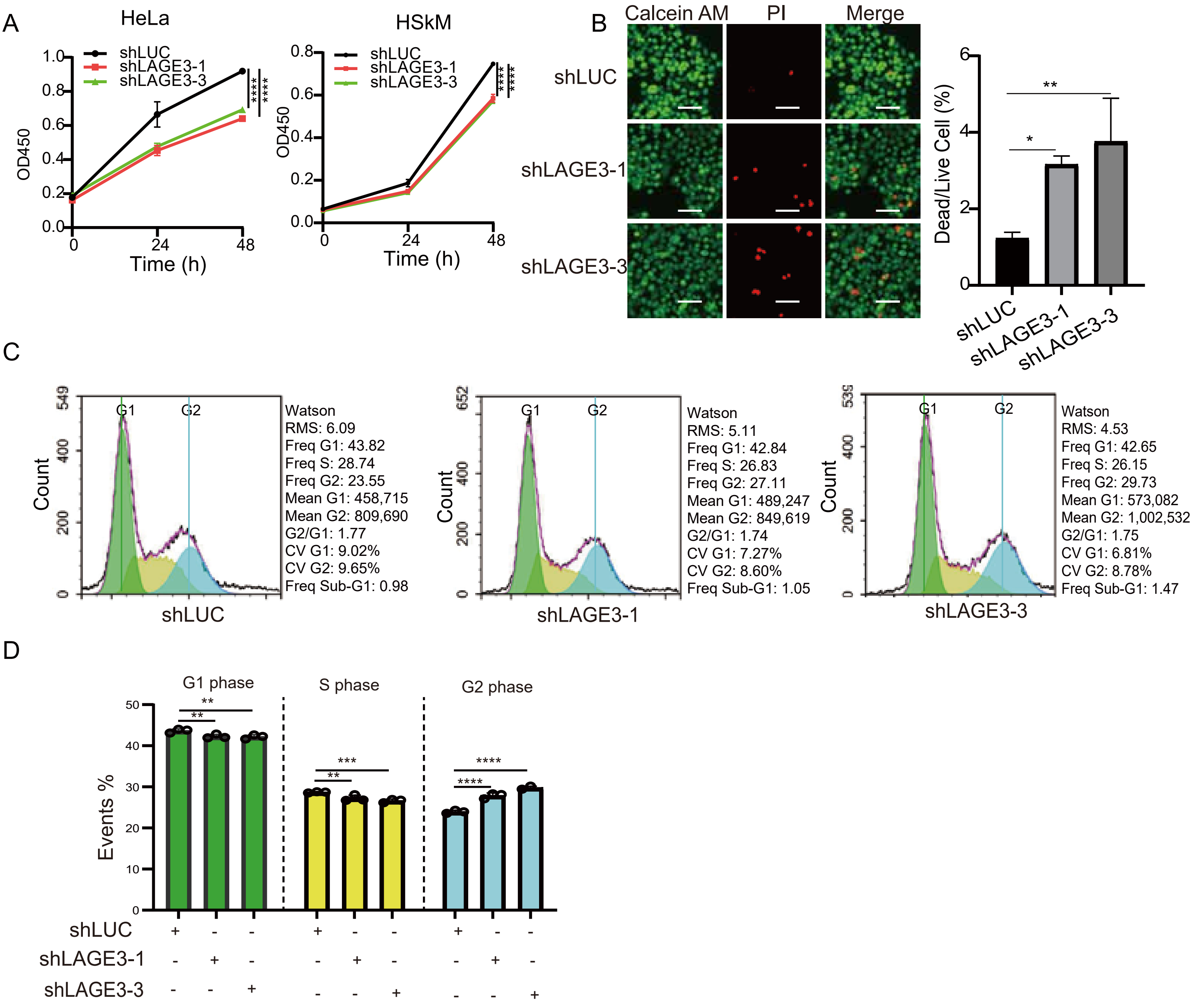

A
HeLa
shLUC
shLAGE3-1
shLAGE3-3
OD450
Time (h)
HSkM
B
Calcein AM
PI
Merge
shLUC
shLAGE3-1
shLAGE3-3
Dead/Live Cell (%)
C
Watson
RMS: 6.09
Freq G1: 43.82
Freq S: 28.74
Freq G2: 23.55
Mean G1: 458,715
Mean G2: 809,690
G2/G1: 1.77
CV G1: 9.02%
CV G2: 9.65%
Freq Sub-G1: 0.98
Watson
RMS: 5.11
Freq G1: 42.84
Freq S: 26.83
Freq G2: 27.11
Mean G1: 489,247
Mean G2: 849,619
G2/G1: 1.74
CV G1: 7.27%
CV G2: 8.60%
Freq Sub-G1: 1.05
Watson
RMS: 4.53
Freq G1: 42.65
Freq S: 26.15
Freq G2: 29.73
Mean G1: 573,082
Mean G2: 1,002,532
G2/G1: 1.75
CV G1: 6.81%
CV G2: 8.78%
Freq Sub-G1: 1.47
Count
D
G1 phase
S phase
G2 phase
Events %
shLUC
shLAGE3-1
shLAGE3-3

Supplemental Figure 1. LAGE3 knockdown reduced cell proliferation and survival.

A. CCK-8 assay analysis of the proliferation of LAGE3-deficient HeLa and HSkM cells. The data were collected from three independent experiments. Statistical analysis was performed with two-way ANOVA, $^{**}P < 0.01$, $^{****}P < 0.0001$. Error bars: SEM.

B. The cell viability of LAGE3-deficient cell was measured with Calcein-AM/PI cell kit. The data were collected from three independent experiments. Statistical analysis was performed with one-way ANOVA, $^{*}P < 0.05$, $^{**}P < 0.01$. Error bars: SEM, scale bar: 50 μm.

C-D. LAGE3 knockdown arrested cell cycle at G2-M phase by flow cytometry analysis. The data were collected from three independent experiments. Statistical analysis was performed with one-way ANOVA, $^{*}P < 0.05$, $^{**}P < 0.01$. Error bars: SEM.

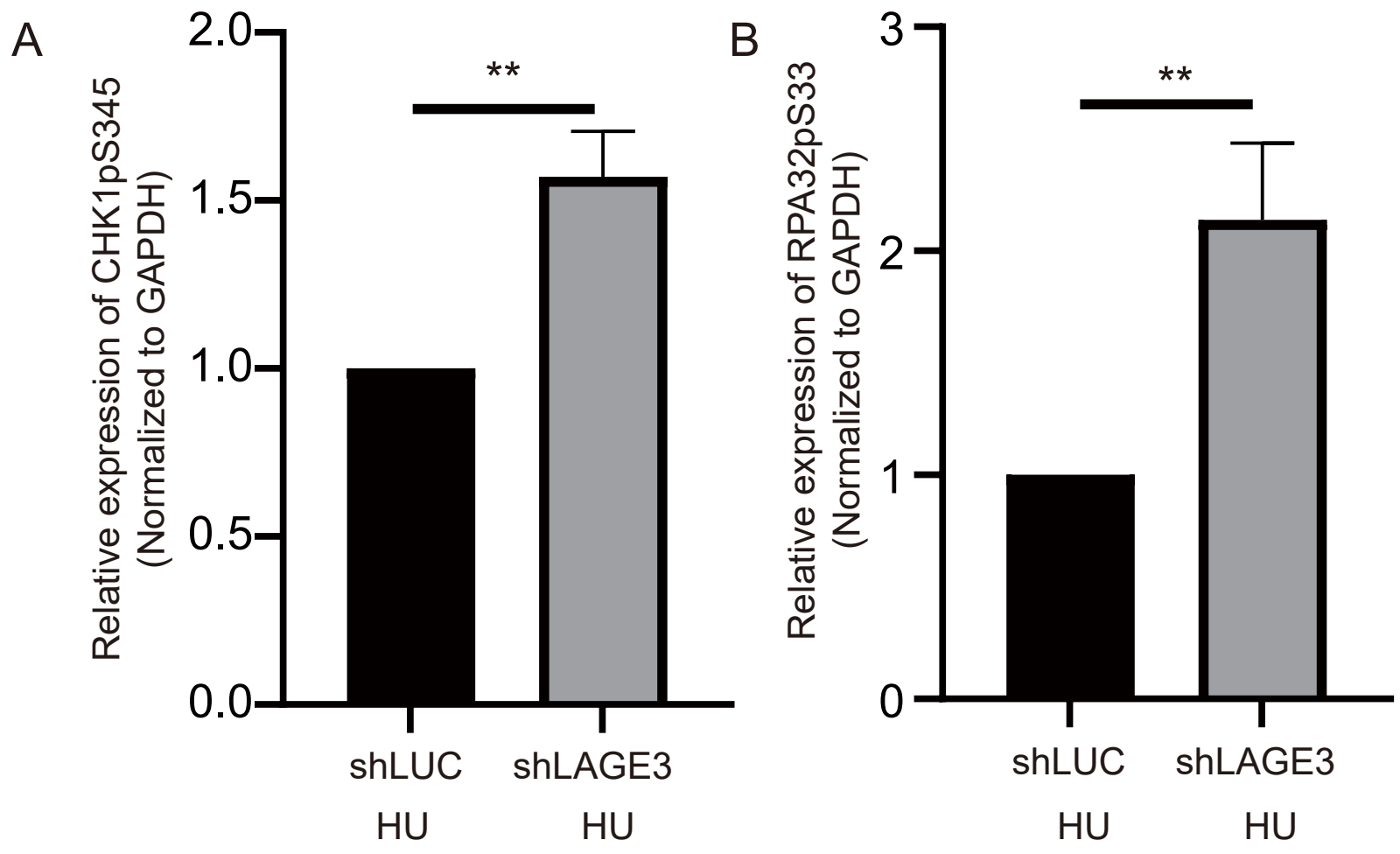
A
B
Relative expression of CHK1pS345
(Normalized to GAPDH)
Relative expression of RPA32pS33
(Normalized to GAPDH)
2.0
1.5
1.0
0.5
0.0
3
2
1
0
**
**
shLUC
HU
shLAGE3
HU

Supplemental Figure 2. Quantification of CHK1pS345 (A) and RPA32pS33 (B) expression under replication stress in LAGE3 knockdown cells (Related to Figure 2B). The target protein intensity was normalized by GAPDH. The data were collected from three independent experiments. Statistical analysis was performed with unpaired Student's t test, $**P < 0.01$. Error bars: SEM.

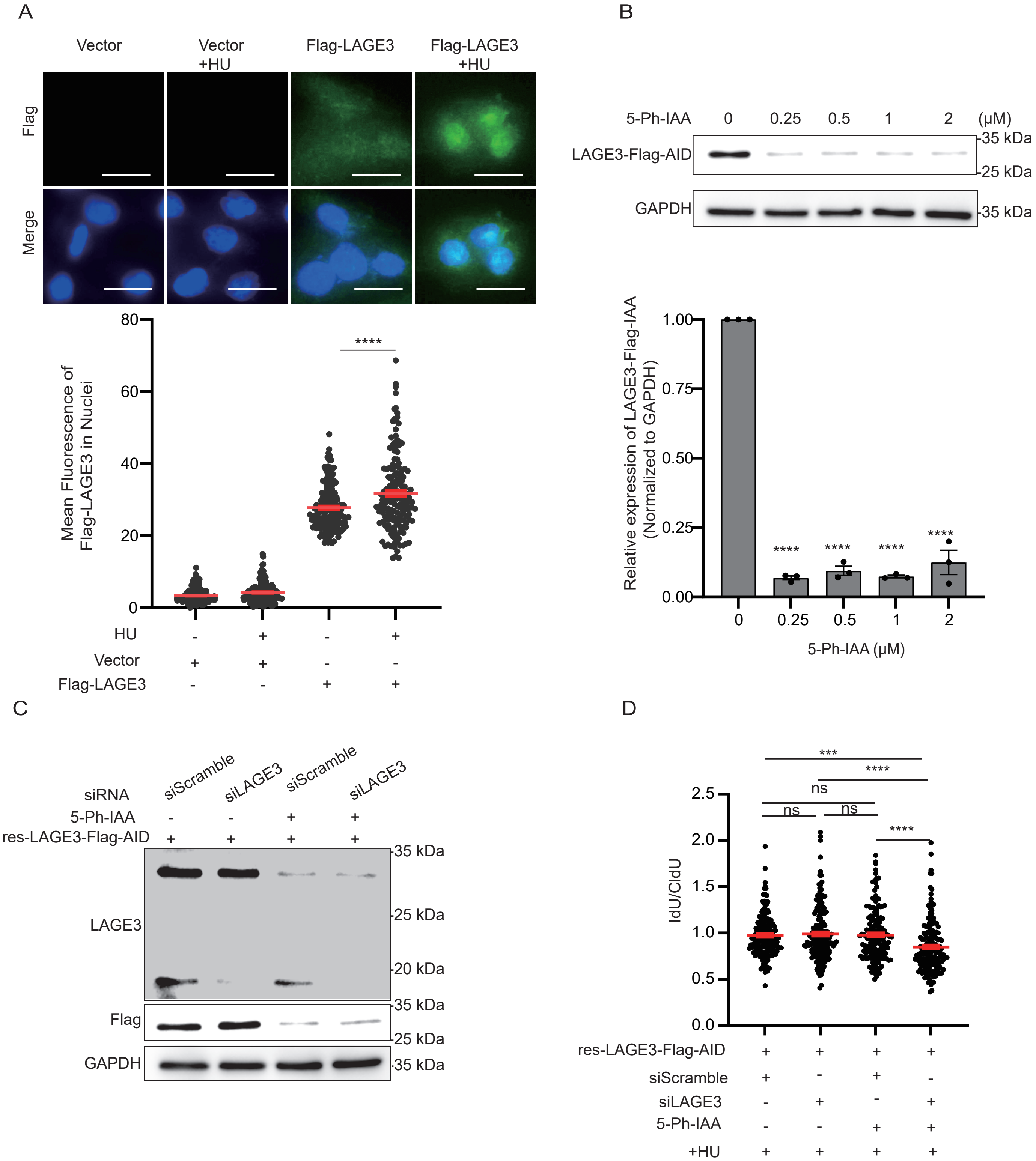
A
Vector
Vector +HU
Flag-LAGE3
Flag-LAGE3 +HU
Flag
Merge
Mean Fluorescence of Flag-LAGE3 in Nuclei
0
20
40
60
80
****
HU - + - +
Vector + + - -
Flag-LAGE3 - - + +
B
5-Ph-IAA 0 0.25 0.5 1 2 (μM)
LAGE3-Flag-AID
GAPDH
35 kDa
25 kDa
35 kDa
Relative expression of LAGE3-Flag-IAA (Normalized to GAPDH)
0.00
0.25
0.50
0.75
1.00
****
****
****
****
0 0.25 0.5 1 2
5-Ph-IAA (μM)
C
siRNA siScramble siLAGE3 siScramble siLAGE3
5-Ph-IAA - - + +
res-LAGE3-Flag-AID + + + +
LAGE3
Flag
GAPDH
35 kDa
25 kDa
20 kDa
35 kDa
25 kDa
35 kDa
D
***
****
ns
ns
ns
****
IdU/CldU
0.0
0.5
1.0
1.5
2.0
2.5
res-LAGE3-Flag-AID + + + +
siScramble + - + -
siLAGE3 - + - +
5-Ph-IAA - - + +
+HU + + + +

Supplemental Figure 3. DNA replication stress triggers LAGE3 accumulating into nucleus and rapid degradation of LAGE3 leads to stalled fork instability.

A. Immunofluorescence showing Flag-LAGE3 accumulation in nucleus under DNA replication stress. Representative images of Flag-LAGE3 immunofluorescence staining are shown. The data were collected from three independent experiments. Statistical analysis was performed with one-way ANOVA, $*P < 0.05$, $**P < 0.01$. Error bars: SEM.

B. HeLa cell with co-expression of Flag-LAGE3-AID2 and osTIR1 (F74G) were treated with the indicated concentration of 5-ph-IAA for 6 h. Western blot showing the degradation efficiency of Flag-LAGE3-AID2 and quantification. The data were collected from three independent experiments. Statistical analysis was performed with one-way ANOVA, $*P < 0.05$, $**P < 0.01$. Error bars: SEM.

C. Establishment of a cellular system to rapidly degrade LAGE3 using AID2 and siRNA targeting endogenous LAGE3 and the efficiency was examined by western blot.

D. DNA fiber analysis indicates rapid degradation of LAGE3 resulting in stalled fork instability under replication stress. The data were collected from three independent experiments. Statistical analysis was performed with one-way ANOVA, $***P < 0.001$, $****P < 0.0001$. Error bars: SEM.

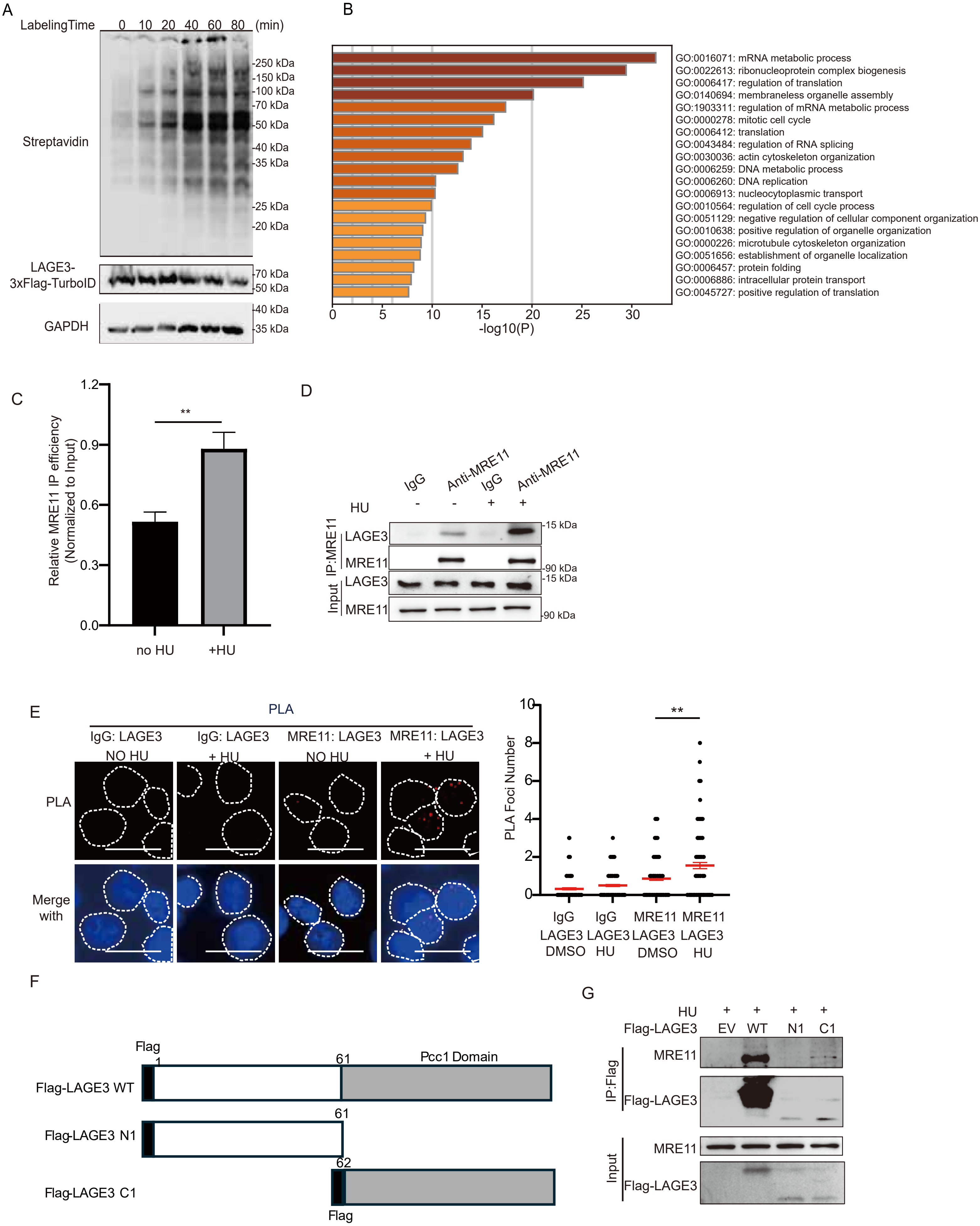
A
LabelingTime 0 10 20 40 60 80 (min)
Streptavidin
-250 kDa
-150 kDa
-100 kDa
-70 kDa
-50 kDa
-40 kDa
-35 kDa
-25 kDa
-20 kDa
LAGE3-
3xFlag-TurboID
-70 kDa
-50 kDa
GAPDH
-40 kDa
-35 kDa
B
GO:0016071: mRNA metabolic process
GO:0022613: ribonucleoprotein complex biogenesis
GO:0006417: regulation of translation
GO:0140694: membraneless organelle assembly
GO:1903311: regulation of mRNA metabolic process
GO:0000278: mitotic cell cycle
GO:0006412: translation
GO:0043484: regulation of RNA splicing
GO:0030036: actin cytoskeleton organization
GO:0006259: DNA metabolic process
GO:0006260: DNA replication
GO:0006913: nucleocytoplasmic transport
GO:0010564: regulation of cell cycle process
GO:0051129: negative regulation of cellular component organization
GO:0010638: positive regulation of organelle organization
GO:0000226: microtubule cytoskeleton organization
GO:0051656: establishment of organelle localization
GO:0006457: protein folding
GO:0006886: intracellular protein transport
GO:0045727: positive regulation of translation
0 5 10 15 20 25 30
-log10(P)
C
Relative MRE11 IP efficiency
(Normalized to Input)
1.2
0.9
0.6
0.3
0.0
**
no HU
+HU
D
IgG
Anti-MRE11
IgG
Anti-MRE11
HU - - + +
IP:MRE11
LAGE3
MRE11
Input
LAGE3
MRE11
-15 kDa
-90 kDa
-15 kDa
-90 kDa
E
PLA
IgG: LAGE3 NO HU
IgG: LAGE3 + HU
MRE11: LAGE3 NO HU
MRE11: LAGE3 + HU
PLA
Merge with
PLA Foci Number
10
8
6
4
2
0
**
IgG LAGE3 DMSO
IgG LAGE3 HU
MRE11 LAGE3 DMSO
MRE11 LAGE3 HU
F
Flag
1
61
Pcc1 Domain
Flag-LAGE3 WT
61
Flag-LAGE3 N1
62
Flag-LAGE3 C1
Flag
G
HU + + + +
Flag-LAGE3 EV WT N1 C1
IP:Flag
MRE11
Flag-LAGE3
Input
MRE11
Flag-LAGE3

Supplemental Figure 4. LAGE3 physically interacts with MRE11.

A. Time-course analysis of TurboID-mediated biotin labeling efficiency in Flag-TurboID-LAGE3 expressing cells. 500 μM biotin were used in this assay.

B. GO term enrichment of biotinylated proteins identified in Flag-TurboID-LAGE3 expressing cells in response to replication stress.

C. Quantification of CoIP efficiency of Flag-LAGE3 with endogenous MRE11(related to Fig. 4D). The data were collected from three independent experiments. Statistical analysis was performed with unpaired Student's t test, **$P < 0.01$. Error bars: SEM.

D. CoIP analysis of the interaction of endogenous LAGE3 and MRE11.

E. PLA analysis of the interaction of endogenous LAGE3 and MRE11. Representative images of PLA foci in nuclei are shown. The data were collected from two independent experiments. Statistical analysis was performed with one-way ANOVA, **$P < 0.01$. Error bars: SEM.

F. Schematic of the Flag-tagged N-terminal domain and the C-terminal domain of LAGE3.

G. Co-IP showing the N- and C-terminal domain of LAGE3 to interact with endogenous MRE11.

Supplemental Fig.5

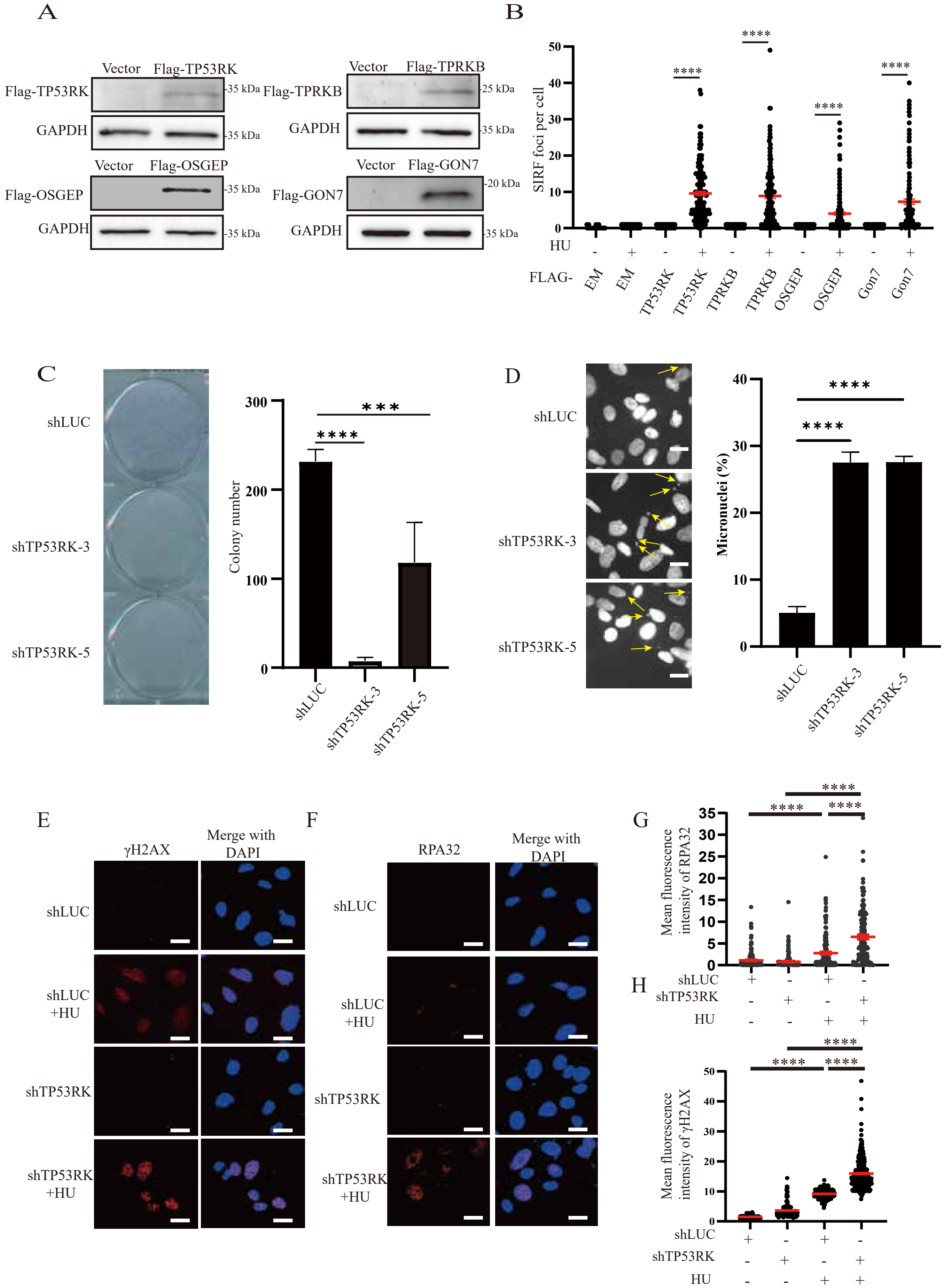

Supplemental Figure 5. Other subunits of KEOPS complex localize on stalled forks.

A. Western blot showing the expression of Flag-TP53RK/TPRKB/OSGEP/GON7 in U2OS cell line.

B. Quantification of Flag-TP53RK/TPRKB/OSGEP/GON7 SIRF foci. The number of SIRF foci per cell was quantified. Mean value was labeled. At least 100 cells were quantified in each sample. The data were collected from two biological repeats and statistical analysis was performed with one-way ANOVA, ****$P < 0.0001$. Error bars: SEM.

C. Colony formation shows that TP53RK deficiency suppresses cell proliferation. Cell colonies were quantified from three independent experiments. P values were calculated by one-way ANOVA analysis, ***$P < 0.001$, ****$P < 0.0001$. Error bars: SEM.

D. Micronuclei were stained with DAPI in TP53RK-depleted cells. Scale bar: 20 μm. The percentage of cells with micronuclei was quantified from three independent experiments. P values were calculated by one-way ANOVA analysis, ****$P < 0.0001$.

E-H. γH2AX and RPA32 immunofluorescence staining were performed in TP53RK-deficient cells under DNA replication stress. The fluorescence intensity for individual cell was quantified. The data were collected from three independent experiments. Scale bar: 20 μm. P values were calculated by one-way ANOVA analysis, ***$P < 0.001$. Error bars: SEM.

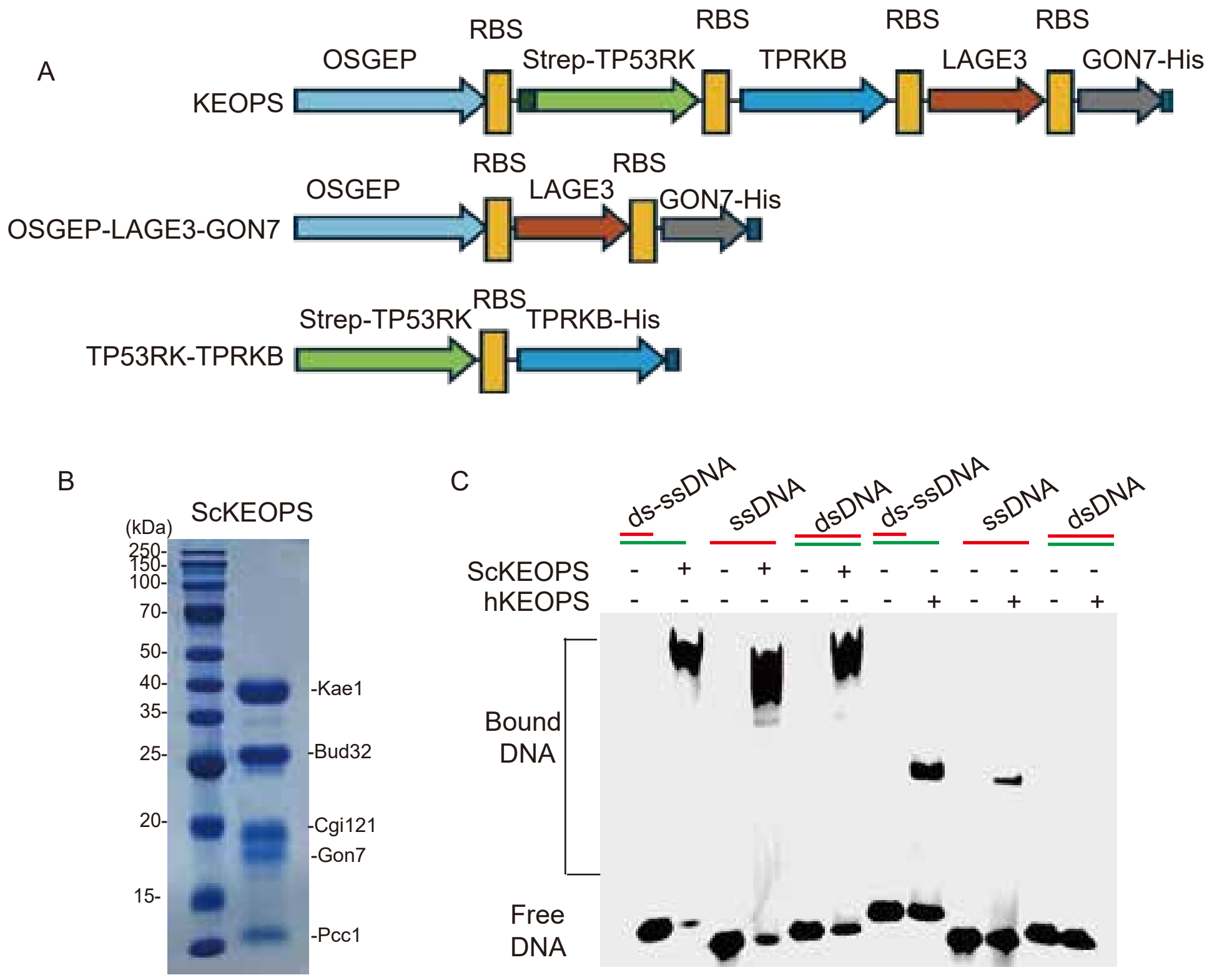

A
KEOPS
OSGEP
RBS
Strep-TP53RK
RBS
TPRKB
RBS
LAGE3
RBS
GON7-His
OSGEP-LAGE3-GON7
OSGEP
RBS
LAGE3
RBS
GON7-His
TP53RK-TPRKB
Strep-TP53RK
RBS
TPRKB-His
B
ScKEOPS
(kDa)
250-
150-
100-
70-
50-
40-
35-
25-
20-
15-
-Kae1
-Bud32
-Cgi121
-Gon7
-Pcc1
C
ds-ssDNA
ssDNA
dsDNA
ds-ssDNA
ssDNA
dsDNA
ScKEOPS
hKEOPS
Bound DNA
Free DNA

Supplemental Figure 6. The validation of KEOPS complex expression and its binding ability of DNA substrates.

A. Schematic of the expression of the human KEOPS complex and its subcomplexes, OSGEP-LAGE3-GON7 and TP53RK-TPRKB (related to Fig. 6A and 6H).

B. Coomassie blue stained SDS-PAGE gel of purified *Saccharomyces cerevisiae* KEOPS complex (ScKEOPS).

C. Cy5.5-labeled DNA substrates were incubated with the human KEOPS complex and ScKEOPS. Samples were analysis by 6% polyacrylamide gel.

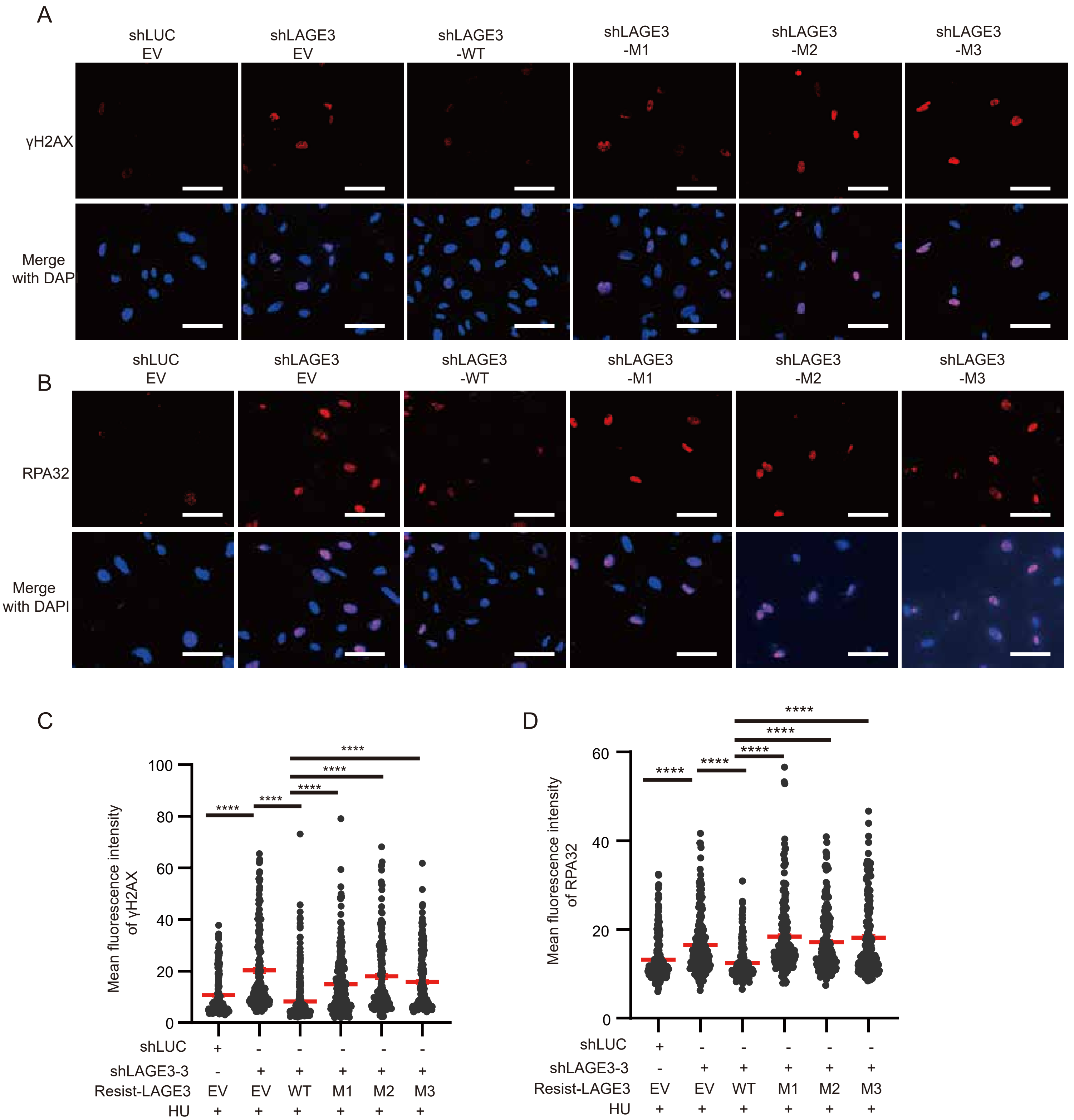

A
shLUC EV
shLAGE3 EV
shLAGE3 -WT
shLAGE3 -M1
shLAGE3 -M2
shLAGE3 -M3
γH2AX
Merge with DAPI
B
shLUC EV
shLAGE3 EV
shLAGE3 -WT
shLAGE3 -M1
shLAGE3 -M2
shLAGE3 -M3
RPA32
Merge with DAPI
C
Mean fluorescence intensity of γH2AX
0
20
40
60
80
100
****
shLUC + - - - - -
shLAGE3-3 - + + + + +
Resist-LAGE3 EV EV WT M1 M2 M3
HU + + + + + +
D
Mean fluorescence intensity of RPA32
0
20
40
60
****
shLUC + - - - - -
shLAGE3-3 - + + + + +
Resist-LAGE3 EV EV WT M1 M2 M3
HU + + + + + +

Supplemental Figure 7. GAMOS-associated LAGE3 mutations led to the damaged DNA accumulation.

(A-B) γH2AX and RPA32 immunofluorescence staining were performed in HeLa cells with LAGE3 knockdown and RNAi-resistant LAGE3 WT and mutations expression with concurrent knockdown of endogenous LAGE3. All the samples were treated with 4 mM HU for 6 h.

(C-D) The fluorescence intensity for individual cell was quantified from three independent experiments. Scale bar: 50 μm. P values were calculated by one-way ANOVA analysis, $^{*}P < 0.05$, $^{**}P < 0.01$, $^{***}P < 0.001$. Error bars: SEM.
